# Simultaneous influence of nanoPSS and photonic crystal on light extraction in AlGaN 304nm UVB LEDs

**DOI:** 10.1038/s41598-025-03746-6

**Published:** 2025-06-06

**Authors:** M. Ajmal Khan, Eriko Matsuura, Yukio Kashima, Hideki Hirayama

**Affiliations:** RIKEN Pioneering Research Institute (PRI), 2-1 Hirosawa, Wako, Saitama, 351-0198 Japan

**Keywords:** Ultraviolet-B light-emitting diode (UVB LED), AlGaN, Light extraction efficiency (LEE), Finite-difference time-domain method (FDTD), Bragg’s condition; plane-wave-expansion (PWE) method, Photonic band gap (PBG), Micro-patterned sapphire substrate (microPSS), Nano-patterned c-plane Sapphire substrate (nanoPSS), Pulsed source, Femtosecond (fs), Applied optics, Lasers, LEDs and light sources, Optical materials and structures, Optical physics, Optical techniques

## Abstract

The external-quantum efficiency (EQE) of AlGaN-based ultraviolet-B light-emitting diodes (UVB LEDs) has achieved a world record value of 9.6% on wafers but suffers from a low light extraction efficiency (LEE) of < 15%, notably lower than that of the LEE of InGaN blue LEDs (> 89%). This study employed the finite-difference time-domain (FDTD) method to explore how micro-patterned c-plane Sapphire substrates (microPSS) or nano-patterned c-plane Sapphire substrates (nanoPSSs) and reflecting photonic crystals (R-PhCs) influence light scattering in flip-chipped AlGaN-based UVB LEDs, with or without an Al-side reflector. First, various microPSS and nanoPSS shapes (Pillar-like and Hole-like) were analysed by the FDTD to optimise the pitch (a), diameter (d), height (h), and diffraction order (m) under Bragg’s condition. The nanoPSS were found most effective for UVB LEDs at an emission peak of 304 nm with cylindrical Hole-like nanoPSS (m = 10, d = 596 nm, a = 746 nm, h = 500 nm, R/a = 0.38), (R is the radius of the holes of the nanoPSS or PhC) improving LEE enhancement to the maximum possible value of approximately 18%. Next, an Al-side reflector was introduced to evaluate the combined impact of optimised nanoPSS and R-PhC (Hole-like) on theoretical light extraction. Parameters (m = 3; h = 150 nm; R/a = 0.40) applied in p-GaN or p-AlGaN contact layers boosted light extraction to approximately 148% or 150% (with an Al-side reflector) and approximately 120% (without an Al-side reflector), marking significant theoretical and experimental advancements in AlGaN UVB LED efficiency.

## Introduction

Aluminium (Al)-rich and Al-moderate based semiconducting aluminium gallium nitride (AlGaN) materials are essential for developing clean and safe deep-ultraviolet (DUV) light-emitting diodes (LEDs) and ultraviolet-B (UVB) LED panels operating in the emission wavelengths range of 210–320 nm^1–10^. Smart, eco-friendly, and high-power AlGaN-based UV LED, photonic crystal (PhC) UV LED, and PhC-based tunnel junction DUV LEDs with enhanced light extraction efficiency modules have attracted increasing attention owing to their various applications. These applications range from water sterilisation, air disinfection, surface disinfection, and other medical sterilisation applications, such as surgical operations and surgical instruments to provide preventive measures against various bacteria, fungi, and viruses, including Severe Acute Respiratory Syndrome Coronavirus 2 (SARS-CoV-2)^4–6^. This UV technology aims to replace toxic mercury UV lamps, aligning with the Minamata Convention (2020)^4,7,10^. Semiconducting AlGaN-based UVB LED light sources are crucial for agricultural, security, communication, and environmental applications^1–4,8–15^. Narrow-band (NB) UVB light sources centred at 311 nm are used for cancer immunotherapy, treating conditions including vulgaris, psoriasis, and atopic dermatitis, and enhancing plant phytochemicals^8–10^. In addition, the UVB emitters centred at a slightly shorter wavelength of 294 nm are suitable for preventing plant diseases, such as tomato mosaic virus (TOMV), fungal disease of powdery mildew in strawberry, and producing vitamin D_3_ in the human body^10,11^. Clean AlGaN UVB and UVC LED light sources are essential for replacing the toxic chemicals and pesticides in the farming industry. These light sources can be widely employed to inactivate bedbugs, mould spores, and combat various infections in plants and vegetables, without harming nature and air quality^2–4,8–14^. However, the performance of AlGaN-based UVB LEDs^8–10,15^ and UVC LEDs^12^ are significantly lower than InGaN blue LEDs^16^.

We faced several challenges with UVC and UVB LED technologies at both the materials and device levels. Achieving high crystal quality in the AlN template on c-plane Sapphire and the n-AlGaN buffer layer beneath the multi-quantum wells (MQWs) of UVB LEDs, with the utilisation of a transparent p-AlGaN hole-source layer (HSL), is crucial for achieving high internal-quantum efficiency (IQE) and light-extraction efficiency (LEE)^8–10,12^. Previously, the choice and quality of the AlN template on microPSS or nanoPSS were found effective by several research groups, including our Laboratory, in achieving high EQE, IQE, and improved LEE in UV emitters^12,14,15^. Lee et al. used nano-patterned AlN/sapphire substrates to enhance the light power of the DUV LED by 67%^17^. However, the low LEE in MQWs grown on c-plane Sapphire substrates is primarily owing to a higher proportion of transverse magnetic (TM)-polarised light compared to transverse electric (TE)-polarised light emission in UV emitters with high AlN-mole-fraction of 0.80 in MQWs on c-plane Sapphire substrates^3,18^. The TE-mode emission is usually higher than the TM-mode emission in the AlGaN MQWs of UVB LEDs with a low AlN mole fraction of 0.40–0.45^8–10^. Shin et al.^5^ demonstrated high external-quantum efficiency (EQE) and wall-plug efficiency (WPE) using nanowire photonic crystal structures in AlGaN DUV LEDs. The LEE is known to be deteriorated by a highly UV absorptive thick p-GaN contact layer^19^, by total internal reflection (TIR) and Fresnel’s loss, arising from the large difference in the refractive indices between air and the AlGaN layer^20^. Numerous studies have reported on the suppression of threading dislocations (TDs) and enhancement of LEE in Al-rich AlGaN layers grown on a c-plane Sapphire substrate using migration-enhanced metal–organic chemical vapour deposition (ME-MOCVD)^17,21,22^. Oder et al.^6^ demonstrated significant improvements in optical power using PhC in UVA and blue LEDs. Wang et al. demonstrated the potential of epitaxial lateral overgrowth (ELO) of the AlN layer on nanoPSS, significantly enhancing IQE to 85%^23^. Kim et al. found that conical PhCs are suitable for enhancing LEE from high refractive index materials^24^. In other LEDs, using PhC enhances the luminescent colour and directional radiation angle for lighting technology^25^. Finite-difference time-domain (FDTD) simulation models have been used to simulate and compare conventional DUV LED and nanophotonic crystal (NPhC)-based DUV LED devices^26^, where LEE of the NPhC-based device was significantly enhanced by 46% and 90% for TE and TM modes, respectively. However, nanoPSS, PhC, and NPhC were unused in the UVB-LED devices by our group^8–10^ and we only used either microPSS^14^ or PhC in UVC LEDs^12^. In our case, the EQE of AlGaN-based UVB LEDs is restricted to 4% on wafers with significantly low LEE of 6–7% due to a low reflective Ni/Au p-electrode and highly UV absorptive p-GaN contact layer^10,12^. However, in the revised experimental UVC LED using reflecting PhC in p-AlGaN contact layer and highly reflective p-electrode the LEE enhancement was significantly improved to 125% (with LEE ≤ 25%)^12^. Additionally, the EQE of our experimental DUV LEDs grown on microPSS has reached a benchmarked value of 20% owing to improved LEE, attributable to the lens-like structure, microPSS, and a highly reflecting p-electrode (Rh) on transparent p-AlGaN contact layer as well as due to the reduced TDs caused by microPSS^14^. LEE enhancement due to microPSS was not reported by Takano et al. given in Ref^14^. In our UVB LED quite low LEE < 15%^10^ and improved IQE ≈ 54–57%^15^, was realized. As a result, after introducing transparent Al-graded p-type multi-quantum-barrier electron-blocking-layer (Al-graded p-MQB EBL) and Al-graded p-AlGaN HSL for the generation and injection of 3D holes in the MQWs with highly reflective p-electrode (Ni/Al) yielded a benchmarked EQE of 9.6% on wafer under continuous-wave (CW) operation at RT^10^. However, the UVB LED devices of our group and other groups are found inferior to UVC LED^12,14^ and blue LED counterparts^16,27^ due to the low LEE.

Moreover, the simultaneous effect of the nanoPSS, and R-PhC in UVB, and DUV LED devices were not studied before^8–10,12,14^. This study examined the simultaneous influence of an optimised Hole-like nanoPSS and R-PhC on the LEE enhancement of flip-chipped surface-mounted device (SMD) AlGaN-based UVB LED using Fujitsu “Poynting for Optics”. First, the influences of different Pillar-like and Hole-like shapes of microPSS and nanoPSS on the light extraction in UVB LEDs were studied. Subsequently, FDTD and the plane-wave-expansion (PWE) methods were employed to examine the influence of the best Hole-like nanoPSS with a 10 nm-thick p-AlGaN HSL and 140 nm-thick p-GaN contact layer (or p-AlGaN contact layer) on light extractions. Finally, we examined the simultaneous influence of Hole-like nanoPSS and PhC in the p-GaN contact layer of UVB LED (Ga-LED) or PhC in the p-AlGaN contact layer on the light extraction in UVB LEDs (Al-LED) either with an Al-side reflector or without an Al-side reflector. This study focused on the integration of nanoPSS and PhC in the experimental UVB LEDs to achieve efficient light extraction and suppress TDs in the active region.

## Numerical methods

**Fig. 1:**
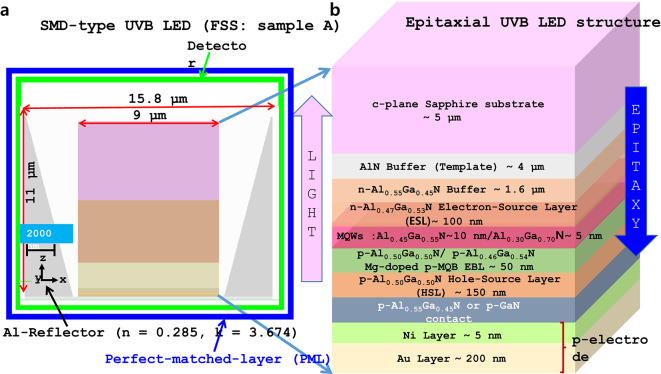
(**a**) Schematic diagram of FDTD computational model and side bird-view of simulated SMD type UVB LED (sample A), where PMLs are employed for absorption boundary condition of FDTD simulation. The detection plane for extracted light is indicated here. (**b**) Schematic cross-sectional view of FDTD computational models for flip-chipped UVB LEDs, with layer-by-layer structure of AlGaN/GaN.

In this study, we assumed that AlGaN-based SMD-type UVB LED structure is grown on microPSS or nanoPSS and coupled with PhC either in p-GaN contact layer (Ga-LED) or in p-AlGaN contact layer (Al-LED), as shown in Fig. 1a. First, the type and size of nanoPSS for UVB LED were investigated. The reference UVB LED structure (sample A) was briefly grown on a 4 μm-thick AlN template, overgrown on 5 μm-thick c-plane Sapphire substrates (without micro or nanoPSS), as shown in Fig. 1a,b. The complete layer-by-layer structure of the reference UVB LED consists of a 1.6 μm-thick Si-doped n-AlGaN buffer layer, a 200 nm-thick Si-doped n-AlGaN electron-source layer (ESL), followed by approximately 4 nm-thick AlGaN-based quantum-well (QW) and a 10 nm-thick lightly Si-doped n-AlGaN quantum-well barrier (QWB) of threefold MQWs structure, as shown in Fig. 1b. Similar information has been provided in Table 1. The *p*-side of the reference UVB LED was composed of a Mg-doped multi-quantum barrier electron-blocking layer (*p*-MQB EBL), which consists of 50 nm-thick lightly Mg-doped p-AlGaN (EBL-I and EBL-II) and relatively insignificant bandgap AlGaN (Valley) structure, an Mg-doped 150 nm-thick p-AlGaN HSL, including either p-GaN contact layer or p-AlGaN contact-layer, as shown in Fig. 1b. Regarding p-electrode, Ni (5 nm) and Au (200 nm) layers are selected, as shown in Fig. 1b. The reflectivity of 34% is chosen for the Ni/Au reflector at 304 nm wavelength emission^8^ figure 1a,b show the simulated.

**Table 1:** Parameters of the epitaxial layers of UVB LED for the FDTD simulation model. To manage the computational load of the FDTD program, the thickness for the optical simulation model is set by the grid size of 5 nm, 10 nm, and 20 nm, respectively.

p-GaN or p-AlGaN contact-layer of UVB LED					FDTD model
Layer	AlN-mole-fraction (x)	Refractive index @ 304 nm	Extinction coefficient	Thickness (nm)	Model thickness (nm)
p-electrode (Au)	-	1.82	1.919	200	200
p-electrode (Ni)	-	1.737	1.993	1	5
p-GaN/p-AlGaN contact	-	2.569	0.374	10	10
p-AlGaN HSL	0.50	2.535		150	150
MQB/barrier	0.50	2.535		12	10
MQB/valley	0.45	2.591		10	10
MQB/barrier	0.50	2.535		16	20
MQB/valley	0.45	2.591		7	10
QW/well	0.30	2.767		4	5
QW/barrier	0.45	2.591		10	10
QW/well	0.30	2.767		4	5
QW/barrier	0.45	2.591		10	10
QW/well	0.30	2.767		4	5
QW/barrier	0.45	2.591		10	10
n-AlGaN ESL	0.47	2.569		100	100
n-AlGaN buffer layer	0.55	2.500		1,600	1,600
AlN buffer layer	-	2.200		4,000	4,000
c-plane sapphire substrate	-	1.810			5,000

schematic structure of SMD-type UVB LED with p-AlGaN contact layer or p-GaN contact layer. The details of other simulation parameters are given in Table 1. TE- or TM-polarised single dipole source with a peak emission wavelength of 304 nm is placed in the MQW region^28^. The lateral dimension for the simulation model is set to 11 µm × 15.8 µm to make a low load for the computational memory, which is less significant than the actual size of UVB. Figure 1a illustrates an LED, where we set the boundary conditions for the four lateral boundaries as Al- metal with high reflectivity such that the limited lateral dimensions can be deemed infinity^12,29^. Next, a perfect-matched layer (PML) was set around the SMD device, which can completely absorb electromagnetic energy^12^. Previously, we used a side reflector in theoretical LED simulations using PML conditions^12^. Additionally, we simulated a scenario without a side reflector, where the boundary condition was set to a PML that absorbs electromagnetic energy completely. Figure 1a illustrates the working detectors. The thickness for the model is set based on grid sizes of 5 nm, 10 nm, and 20 nm because of the load of the FDTD program, ensuring accuracy in calculating LEE. The absorption coefficients of the AlGaN layer, MQWs, and p-GaN layer, respectively, are chosen to be 10 cm^−1^, 1000 cm^−1^, and 170,000 cm^−1^^30,31^. The refractive indices of the c-plane Sapphire substrate, AlN layer, AlGaN layer, and GaN layer, respectively, are chosen to be 1.810, 2.200, 2.569, and 2.600. More refractive indices data details of layer-by-layer UVB-LED structures are given in Table 1.

Regarding the nanoPSS investigation, similar UVB-LEDs were considered. However, different patterning conditions in c-plane Sapphire substrates were introduced, including flat-surface sapphire substrate (FSS)-based LED (sample A), as shown in Fig. 1b, and nanoPSS-based LED (N-LED), as shown in Fig. 3. First, we examined the influences of different shapes and types, including (Cone + Dome)-like and Pyramid-like or Hole-like structures in the microPSS and (Cone + Dome)-like or Hole-like structures in the nanoPSS on the optical performances of the UVB devices. Subsequently, the influence of the most optimised Hole-like nanoPSS on the light extraction of UVB LEDs was optically simulated and presented.

Finally, we examined the simultaneous influence of the most optimised hole-type nanoPSS and R-PhC (refer to R-PhC as a “PhC”) on the theoretical power emission and light extraction in the SMD AlGaN-based UVB LED. Additionally, we examined two different PhC in the p-GaN contact layer, including nanoPSS (Ga-LED) with an Al-side reflector. This includes PhC in the p-AlGaN contact layer, including nanoPSS (Al-LED) either with an Al-side reflector or without an Al-side reflector. The total thickness of the p-AlGaN (20 nm) and p-GaN (140 nm) contact layer was set to 160 nm as the original structure. The remaining structure of Ga-LED is similar to that of the nanoPSS case (N-LED), as shown in Fig. 3. Subsequently, the FDTD method and PWE were employed to examine the influence of the nanoPSS and PhC in 10 nm-thick p-AlGaN HSL and 150 nm-thick p-AlGaN contact layer on light extraction. The remaining structure is similar to that used for the nanoPSS case (N-LED), as shown in Fig. 3. We compared two cases for the transparent p-AlGaN contact layer: one without an Al-side reflector and the second with an Al-side reflector. Finally, we proposed a new optical design for the epitaxial growers of AlGaN UVB emitters in molecular-beam epitaxy (MBE) and MOCVD to simultaneously enhance LEE and reduce TDs in the quantum well.

To determine the nanoPSS structure and PhC for the p-GaN contact and p-AlGaN contact layer cases, the FDTD analysis and following Bragg’s Equation are used as follows:

1 $$m\frac{\lambda }{neff} = 2a$$

Where order (m) stands for the well-known order of light diffraction, and *n*_*eff,*_ λ, and *a* are the effective refractive index, wavelength, and lattice period (pitch), respectively. The Plane-Wave Expansion (PWE) method was employed to investigate the photonic-band gap of the nanoPSS and PhC structure of AlGaN-based UVB-LEDs. Hole-like nanostructures were introduced in the c-plane Sapphire and the nano Hole-arrays were assumed to be filled with AlN material. Figure 4a shows the inset, where n_1_ and n_2_ represent the refractive index of c-plane Sapphire (n_2_ = 1.81) and AlN (n_1_ = 2.2), respectively.

## Results and discussion

**Fig. 2:**
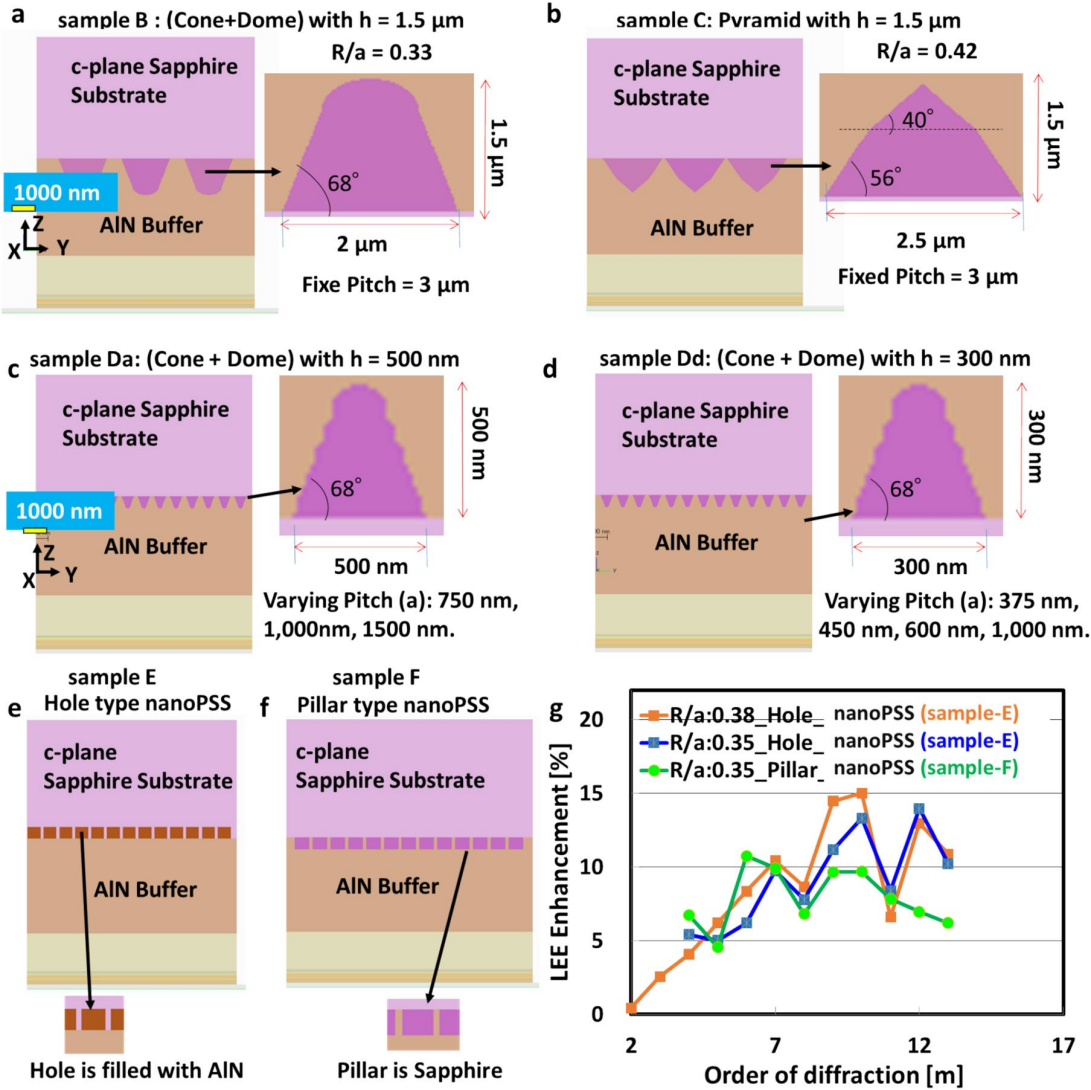
Schematic diagram of FDTD computational domain for microPSS using (**a**) (Cone + Dome)-like (sample B) with height (h) = 1.5 µm, diameter (d) = 2 µm, and pitch (a) = 3µm, (**b**) Pyramid-like (sample C) with h = 1.5 µm, d = 2.5 µm, and a = 3 µm. Schematic diagram of FDTD computational domain for nanoPSS, (**c**). (Cone + Dome)-like nanoPSS (sample Da) with h = 500 nm, d = 500 nm and ‘a’ was allowed to vary between 700 nm – 1500 nm, (**d**). (Cone + Dome)-like nanoPSS structure (sample Dd) with d = 300 nm, h = 300 nm and R/a was allowed to vary between 0.17- 0.33. Computational FDTD models of SMD UVB LEDs, illustrating the cross-section for (**e**) rectangular Hole-like (sample E), (**f**). Pillar-like (sample F) nanoPSS structure, and (**g**) Light extraction enhancement variation of rectangular Hole-like (sample E) and Pillar-like (sample F) nanoPSS as a function of diffraction order (m) by keeping R/a = 0.35 and 0.38, respectively.

A comprehensive study of the influences of microPSS, nanoPSS, and PhC on the optical performances of 304 nm-band UVB LEDs was conducted, as shown in Fig. 2a-g. For microPSS or nanoPSS, similar simulation models of UVB LEDs were used despite various patterning shapes being introduced in the c-plane Sapphire substrates. These patterns included starting FSS, Pyramid-like, (Cone + Dome)-like microPSS and nanoPSS, and Pillar and Hole-like nanoPSS, respectively. These optimisation details of design structures and optical simulation results are given in the Supplementary note. However, herein we first briefly discussed the simulation results of microPSS and nanoPSS on the LEE enhancement in UVB LED using various possible geometries in section"Influence of the microPSS and nanoPSS on the LEE enhancement in UVB LED". Subsequently, the most promising Hole-like structure-based nanoPSS (N-LED) of UVB LED is discussed. Sections"Simultaneous influence of nanoPSS and photonic crystal in p-GaN contact layer on the optical performance of UVB LED“and”Simultaneous influence of nanoPSS and photonic crystal in transparent p-AlGaN contact layer on the optical performance of UVB LED"discuss the simultaneous influence results of the most optimised Hole-like nanoPSS and two different PhCs (Ga-LED and Al-LED) on theoretical light extraction in the SMD-type AlGaN-based UVB LED. Section"Simultaneous influence of nanoPSS and photonic crystal in transparent p-AlGaN contact layer on the optical performance of UVB LED"also compares two cases for the transparent p-AlGaN contact layer: one without a side reflector and the second with an Al-side reflector. First, we will briefly discuss the simulation results of microPSS and nanoPSS on the LEE enhancement in UVB LED using various possible geometries, radius of the hole or pillar (R)/pitch (a) ratios, shapes, sizes, etc. to get the most promising nanoPSS.

### Influence of the microPSS and nanoPSS on the LEE enhancement in UVB LED

This section discusses qualitatively the results of using different shapes and sizes of microPSS or nanoPSS in UVB LEDs at 304 nm emission wavelength, as given in the schematic view of Fig. 2a-g. Firstly, we examined the FDTD simulation of two microPSS either having (Cone + Dome)-like shape (sample B) or Pyramid-like shape (sample C) on the light extraction in UVB LED, as shown in Fig. 2a,b and compared it with the UVB LED on FSS (sample A). Consequently, (Cone + Dome)-like (sample B) yielded LEE enhancement of approximately 7% using R/a = 0.33 (where R is the radius of the holes of the nanoPSS or PhC), diameter (d) = 2.0 µm, height (h) = 1.5 µm, and pitch (a) = 3 µm compared to samples A and C, respectively. Secondly, we examined the FDTD simulation model of (Cone + Dome)-like nanoPSS (sample Da) with fixed h = 500 nm, d = 500 nm, where only pitch (a) could vary between 700–1500 nm, as shown in Fig. 2c,d. Additionally, we examined the influence of relatively insignificant and practical (Cone + Dome)-like nanoPSS (sample Dd) on the light extraction with d = 300 nm and h = 300 nm. The (Cone + Dome)-like nanoPSS-based UVB LED (sample Dd) yielded LEE enhancement of approximately 6% using R/a = 0.33 and a = 450 nm compared to the results of other R/a values. Finally, it was concluded that Pillar-like microPSS and nanoPSS are unsuitable for UVB LEDs because the LEE enhancement were restricted to approximately ∼ 6–7%. The influence of Hole-like nanoPSS structure in UVB LED on the light extraction remained challenging. Thirdly, we simulated and analysed the performances of Hole-like nanoPSS (sample E) and Pillar-like nanoPSS (sample F) structures in the AlGaN UVB LEDs, as shown in Fig. 2e,f, where the diffraction order (m) could vary from 1 to 13. Consequently, a significant improvement in the LEE enhancement of approximately ∼ 15% was achieved in UVB LED using diffraction order (m) in crystal = 10 and R/a = 0.38, as shown in Fig. 2g. Therefore, the light extraction of Hole-like nanoPSS (sample E) was superior to that of the Pillar-like nanoPSS (sample F) using the same R/a = 0.35, as shown in Fig. 2g. More detailed information on the results and analysis of microPSS and nanoPSS can be found in the Supplementary note.

**Fig. 3:**
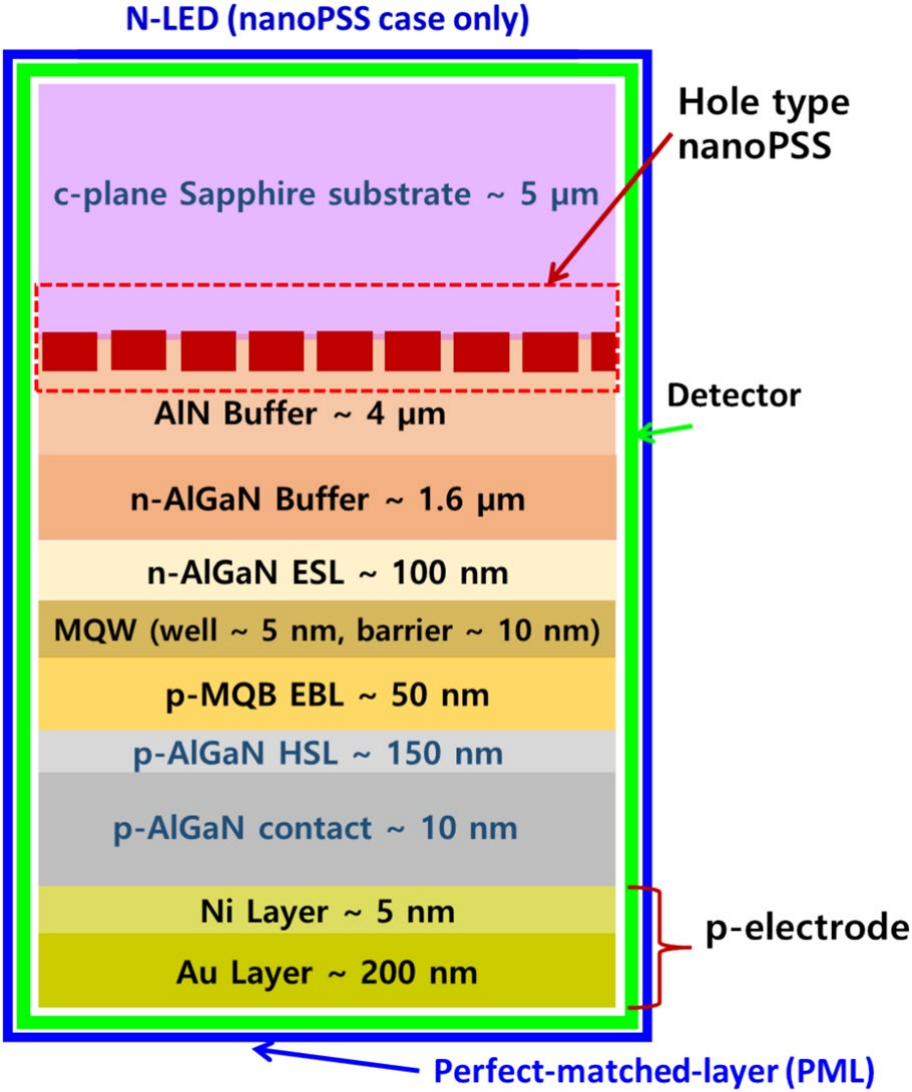
Schematic view of computational models for flip-chipped UVB LEDs (N-LED), with nano-patterned hole distribution in the c-plane Sapphire substrate (nanoPSS) without PhC. The geometrical and structural parameters of the LED are given in the inset.

**Fig. 4:**
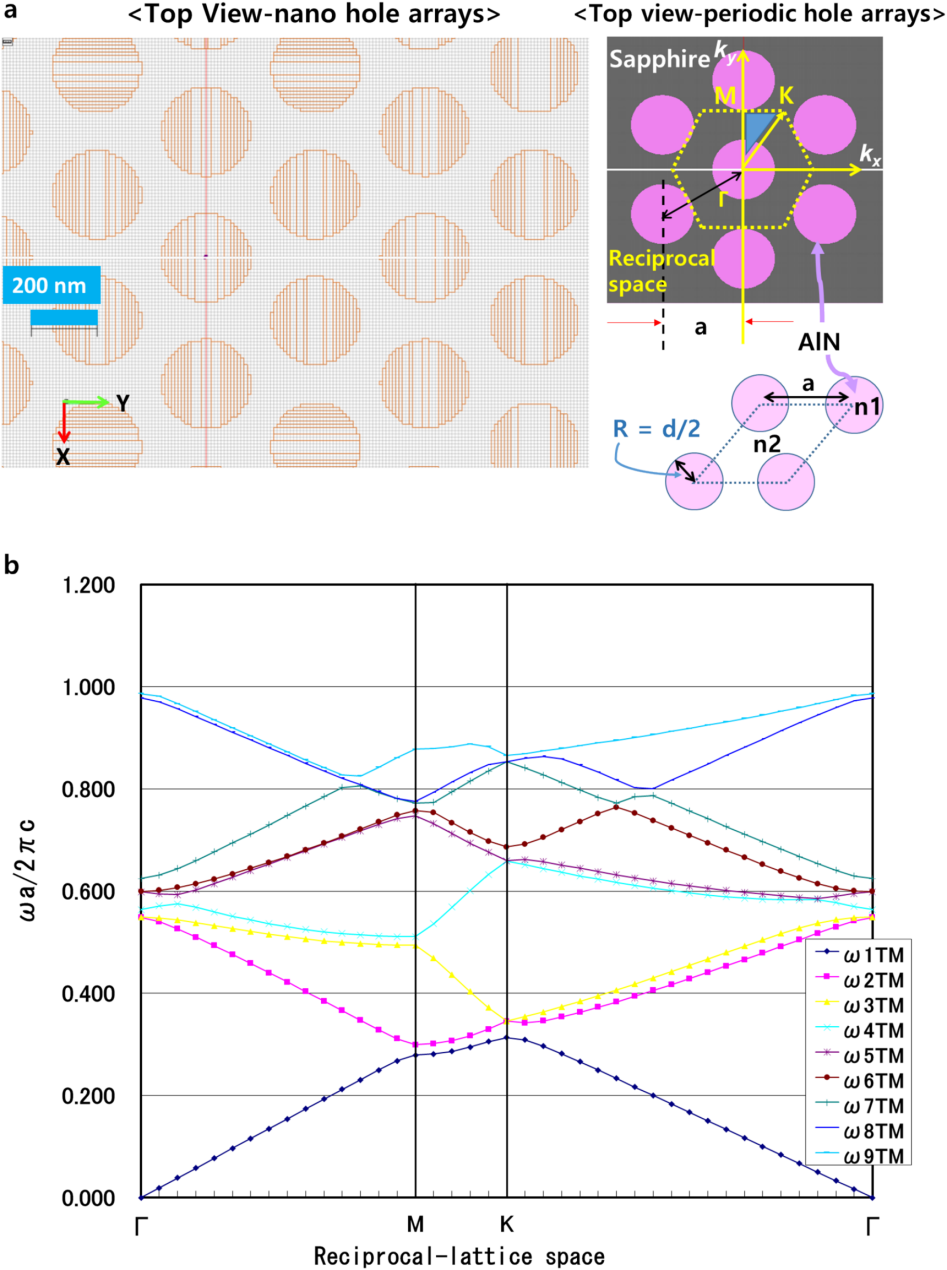
(**a**) Computational models of flip-chipped UVB LEDs with top view of nano-patterned hole distribution in the c-plane Sapphire substrate (FSS-based LED). A magnified view of the nano-patterned hole distribution in the c-plane Sapphire is shown in the inset with the reciprocal space. (**b**) Optical band diagram of a circular Hole-like structure on the surface of c-plane Sapphire substrate.

This study demonstrated that the cylindrical Hole-like nanoPSS structure (sample E) was suitable for practical applications in UVB LED to enhance light extraction. Therefore. in this study, we further refined the cylindrical Hole-like nanoPSS in UVB LED (see Table 2) for enhanced light extraction of emitted light. Moreover, a Hole-like structure was assumed to be filled with AlN, as shown in the inset of Figs. 3 and 4a. The refractive indices of AlN (n_1_ = 2.2) and sapphire (n_2_ = 1.81), respectively, are used in Fig. 4a. This study investigated the influence of the most suitable nanoPSS for UVB wavelength on the light extraction enhancement using a 150 nm-thick p-AlGaN HSL and 10 nm-thick p-AlGaN contact layer, as shown in Fig. 3. Figure 3 shows the side view of the optically simulated nanoPSS-based LED (N-LED) structure. Figure 4a shows the Hole-like structure of the LED, where all the geometrical conditions, including the FDTD computational domain schematic diagram, are given. We used the PWE method^32^ to investigate the optical band diagram of the photonic crystal structure in a circular Hole-like structure on the surface of c-plane Sapphire in the reciprocal space, as shown in Fig. 4a. Figure 4b illustrates the plot of crystallographic directions in the reciprocal-lattice space (M, K, and Γ) of nanoPSS. Figure 4b illustrates the optical band diagram, demonstrating that the optical band gap of nanoPSS for TM-polarisation modes is not observable even in the optimised design of the Hole-like nanoPSS structure of UVB LED.

**Table 2:** R/a values of Hole-like nanoPSS (N-LED) with fixed diffraction order (m) = 10, and h = 500 nm used and only diameter (d) and pitch (a) are varied.

Parameter	Hole type pattern	
R/a	d (nm)	a (nm)
0.25	400	800
0.30	470	784
0.35	536	765
0.38	573	754
0.40	596	746

**Fig. 5:**
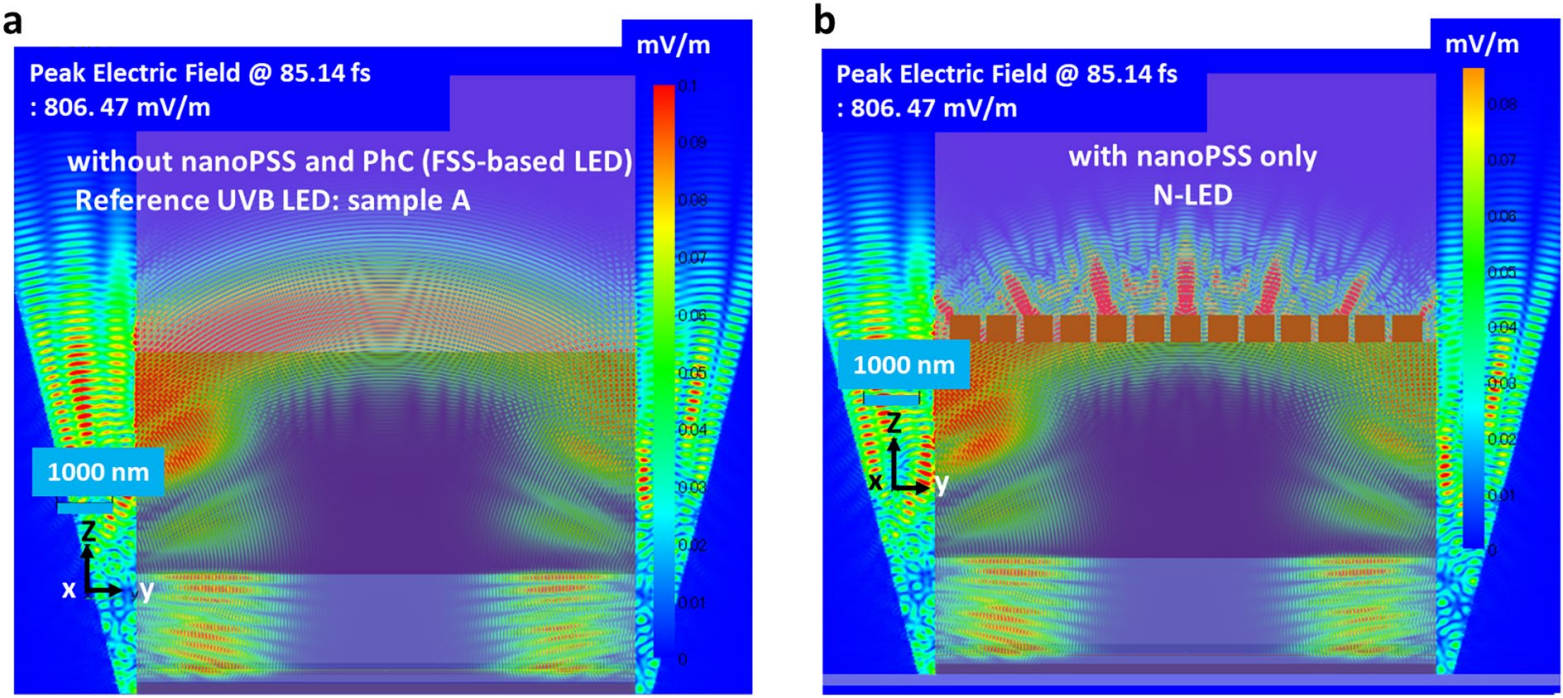
Cross-sectional in-plane electric-field mappings of 304 nm UVB LEDs (**a**) without nanoPSS (FSS-based LED) sample-A, and (**b**) with Hole-like nanoPSS structure-based LED (N-LED) using FDTD method.

The best-optimised nanoPSS pattern with depth (h) = 500 nm and with varying parameters of R/a values between 0.25 and 0.40, using diffraction order (m) = 10 in UVB LED was introduced, as given in Table 2. Figure 5a,b shows the cross-sectional electric-field (E-field) mappings of the AlGaN-based UVB LEDs without nanoPSS (sample A) and with Hole-like nanoPSS structure (N-LED), calculated using the FDTD model for light extraction. The in-plane E-field mappings are calculated at the interface between the AlN and c-plane Sapphire substrate, particularly in a plane where the hole arrays of the nanoPSS are filled with AlN. Table 2 illustrates that the cross-sectional E-field mappings given in Fig. 5b demonstrate that by using a nanoPSS structure with the optimised parameters, the light extraction from the MQWs of UVB LEDs was significantly enhanced compared to the other nanoPSS cases, including FSS-based LED (sample A), as shown in Fig. 5a.

**Fig. 6:**
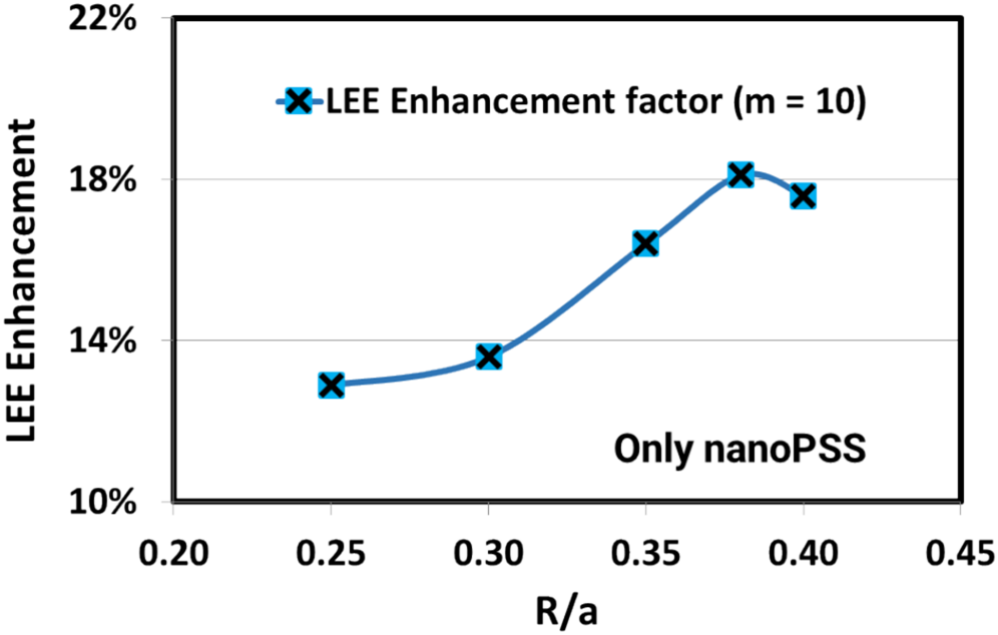
LEE enhancement variation as a function R/a ratio using diffraction order (m) = 10, depth = 500 nm in hole-like nanoPSS-based UVB LED (N-LED).

Figure 6 illustrates the calculated light extraction of a Hole-like nanoPSS-based AlGaN UVB LED (N-LED). The estimated values of LEE enhancement are improved for the nanoPSS using R/a = 0.38, diffraction order (m) = 10, Hole-depth (h) = 500 nm, d = 573 nm, and a = 754 nm, compared to the FSS-based LED (sample A). Regarding the usual FSS-based LED (without nanoPSS and PhC; sample A), the UV light from the QWs propagates equally in all directions, as shown in Fig. 5a, and the UV light from the QWs of N-LED propagates in vertical (guided direction), as shown in Fig. 5b. Table 1 and Table 2 illustrate the structural input parameters used in this Calculation. The LEE enhancement increased as a function of R/a increasing from 0.25 to 0.38, subsequently decreasing beyond R/a = 0.40 value. Consequently, the maximum LEE enhancement > 18% was achieved using only Hole-like nanoPSS by setting R/a = 0.38, m = 10, Hole-depth (h) = 500 nm, d = 573 nm, and a = 754 nm, as illustrated in Table 2 and Fig. 6. To obtain the maximum possible light extraction in UVB LED at an emission wavelength of 304 nm, the periodicity and R/a ratio of the Hole-like nanoPSS in UVB LED are further tuned. Additionally, for a higher diffraction order (m) = 11, 13, the light extraction of a Hole-like nanoPSS-based AlGaN UVB LED (N-LED) is decreasing. Therefore, we choose the diffraction order (m) = 10 for our best nanoPSS to be used in UVB LED for both reduction of dislocations density and light extraction enhancement. The larger R/a of approximately 0.38 yields a superior LEE enhancement of 18.1%, demonstrating that the cylindrical Hole-like nanoPSS structure is superior to the rectangular Hole-like structure (sample E), where the maximum LEE enhancement of 14% is achieved, as shown in Fig. 2g. As the R/a ratio increased beyond 0.40 in N-LED, the light extraction decreased to 15%, as shown in Fig. 6. Therefore, we choose the best nanoPSS design of sample N-LED with diffraction order (m) = 10 for the simultaneous influence of nanoPSS and PhC in UVB LED. Section"Simultaneous influence of nanoPSS and photonic crystal in p-GaN contact layer on the optical performance of UVB LED"discusses the simultaneous influence of the nanoPSS and PhC in the p-GaN contact layer on the optical performance of UVB LED.

### Simultaneous influence of nanoPSS and photonic crystal in p-GaN contact layer on the optical performance of UVB LED

**Fig. 7:**
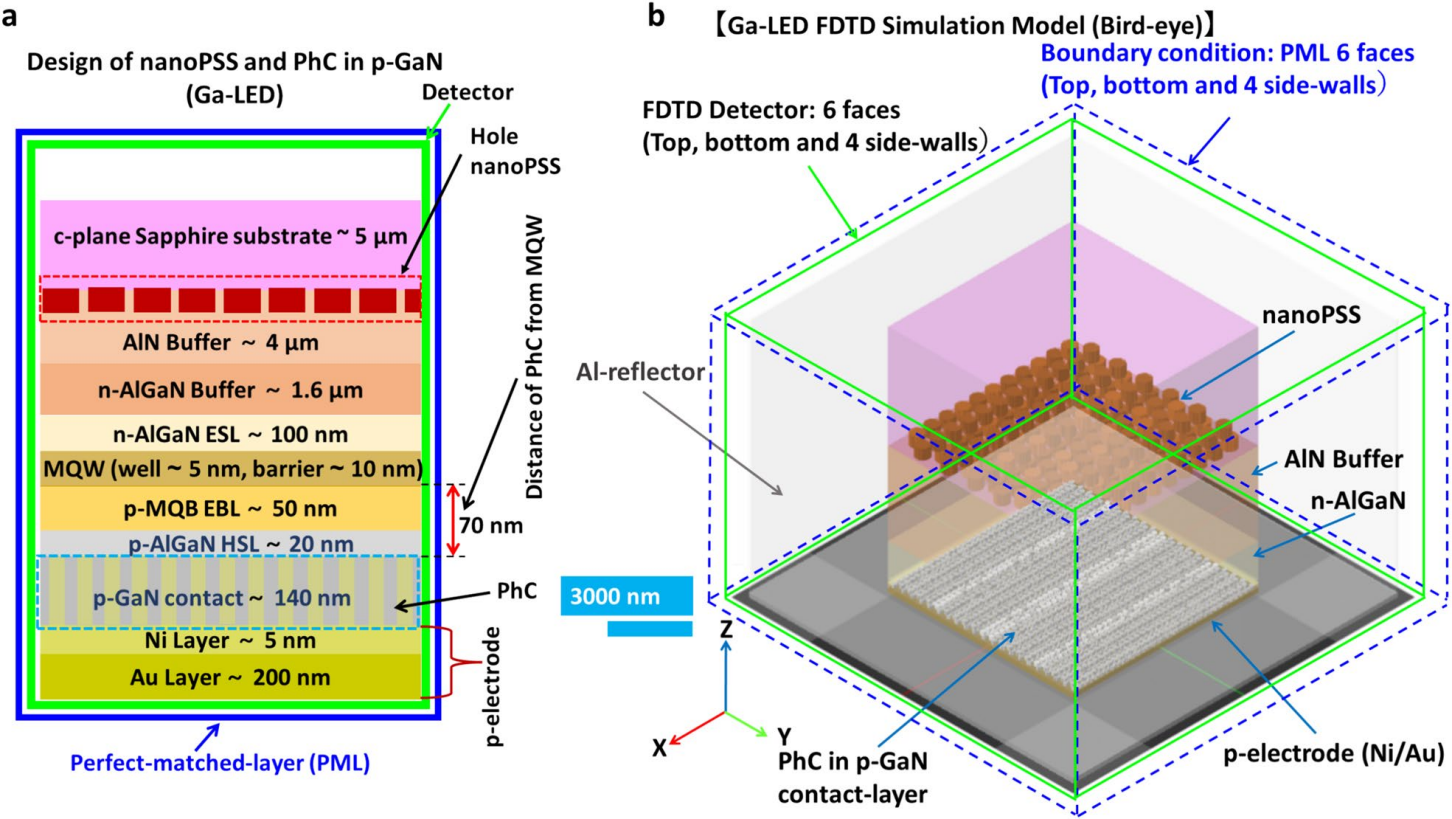
(**a**) Schematic computational models for flip-chip UVB LEDs with hole-like nanoPSS and PhC in p-GaN contact layer (Ga-LED) and (**b**) 3D view of the Al-LED with nano-patterned hole distribution in c-plane Sapphire substrate and PhC in the p-AlGaN contact layer including the PML condition and detectors around the six faces.

In this study, we designed a simulation model for real LED devices (SMD), shown in Fig. 7a,b. Real LED devices (SMD Packaged) often have Au reflectors. However, we use an Al reflector in our devices. Moreover, our SMD device yields diffused reflection with a high reflectivity, approximately equal to the reflectivity of Al. However, the diffused reflection reflector, which is too thick with dozens of microns, poses challenges in the FDTD simulation model. Therefore, we used an Al reflector in this simulation model. The PML boundary condition was set outside the FDTD detectors on six faces (top, bottom, and four side walls), as shown in Fig. 7b. Our simulation model had the FDTD detectors on six faces (top, bottom, and four side walls) outside the LED structure. Figures 7a,b show the simple cross-sectional and 3D bird-eye view of the simulation strategy. However, designing PhC in the p-AlGaN contact layer is challenging compared to the fabrication of PhC in the p-GaN contact layer of UV LEDs. Previously, we could not obtain effective PhC structure in p-AlGaN contact layer surfaces of DUV LEDs because the etching process caused damage to the p-AlGaN contact layer surfaces in PhC when relatively high Al-contents > 70% were used^12^. However, we fabricated a low-damage PhC with suitable R/a values on the surface of the p-AlGaN contact layer when the Al-contents < 70% in the UVC LED by using a nanoimprinting technique and an inductively coupled plasma (ICP) dry-etching processes^12^. The surface damage induced during the dry-etching process can damage the centre for hole carriers owing to the self-compensation effect in high Al-contents UV LEDs. The carrier-injection efficiency (CIE) was significantly reduced owing to the lower hole density in the PhC of the p-AlGaN layer. The IQE of the DUV emission from the QW decreased owing to the low hole injection toward the MQWs emitter caused by the self-compensation effect in the damaged p-AlGaN PhC. For our first few UVC devices, we chose R/a = 0.21 to minimise surface damage by maintaining an insignificant etching area^12^. Higher R/a values were preferable for obtaining high LEE for a specific choice of UV emission wavelength^12^. Regarding the revised experimental DUV LED with PhC in p-AlGaN contact layer using highly reflective Ni (1 nm)/Mg (200 nm) p-electrode, the LEE was significantly enhanced to 20–25% using 60 nm diameter PhC, R/a = 0.4 and maintaining a PhC structure distance from MQW of approximately 60 nm^12^. The LEE enhancement factor strongly depends on the R/a value of PhC and the distance of PhC from the quantum well^12,33^. Therefore, we analysed the UVB-LED optical model by introducing PhC in the p-GaN contact layer without necessitating the p-AlGaN contact layer’s etching. Next, we compared it with the PhC in the pure p-AlGaN contact layer (no p-GaN contact layer case), where etching of p-AlGaN material was challenging but less challenging than the DUV case, in which p-Al_0.70_Ga_0.30_N was used. The UV light absorption in the p-GaN contact layer can be significantly reduced. By fabricating a PhC on the surface of the p-AlGaN/p-GaN (top) contact layer of UVB LED, UV light can be reflected at the PhC p-AlGaN contact layer. Therefore, high hole conductivity and low contact resistance via a p-GaN contact layer can be achieved. Hence, fabricating PhC in the p-AlGaN/p-GaN contact layer yields high LEE in the UVB LED, supporting high WPE. Previously, reflective-PhC surface-emitting lasers could provide high-quality radiation beams in the vertical direction^33^. This method is suitable for obtaining high spontaneous emission reflection from the MQWs of UVB LED in the vertical direction^12^. Therefore, one-directional vertical emission can be obtained by satisfying the lateral Bragg’s conditions and locating the MQWs emitter at a specific position near the R-PhC^12,33^.

**Fig. 8:**
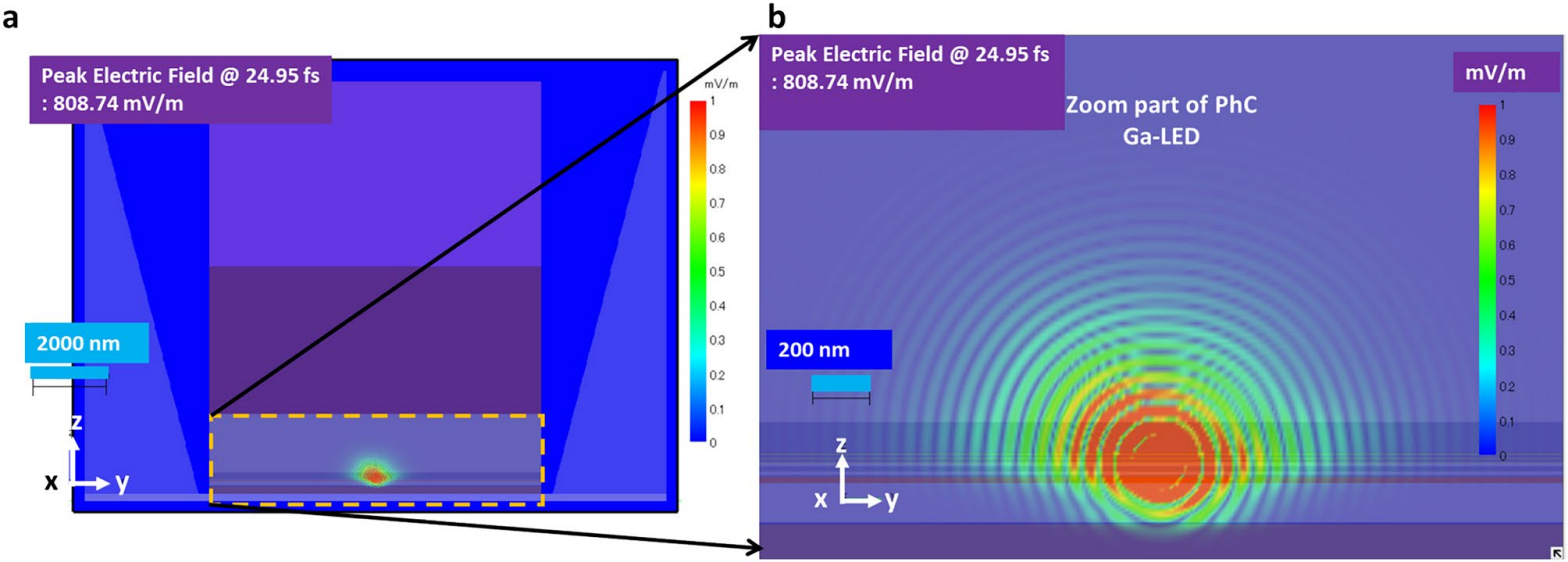
(**a**) Cross-sectional E-field mappings of 304 nm UVB LEDs (Ga-LED) through PhC in the case of Hole-like nanoPSS and PhC, calculated using the FDTD method, and (**b**) Enlarged cross-sectional E-field mappings near the PhC of LED (Ga-LED).

This study examined the simultaneous influence of reflecting photonic crystal periodic structure in the p-GaN contact layer of UVB LED (Ga-LED), as shown in Fig. 7a,b. This includes the best-optimised nanoPSS structure (N-LED), discussed in Section"Influence of the microPSS and nanoPSS on the LEE enhancement in UVB LED". The total thickness of the p-AlGaN HSL and p-GaN contact layer was set to 160 nm as of the original LED structure, as shown in Fig. 7a. The 140 nm-thick p-GaN contact layer is suitable for the oblique deposition of p-electrode (Ni/Au or Ni/Al or Rh or Ru). The 15 nm-thin p-GaN contact layer might be most promising, but the oblique deposition of p-electrode (Ni/Au or Ni/Al or Rh or Ru) might be challenging. The PhC can be set within the p-GaN contact layer. The maximum light enhancement factor was found at 60–70 nm between PhC and MQW (L_QP_). Therefore, the position of the PhC structure is set at 70 nm from MQWs, as shown in Fig. 7a. Tables 1, 2, and 3 illustrate that the remaining structure of the UVB LED is similar to N-LED (nanoPSS), as shown in Fig. 3, and Fig. 4a. We assumed an air-filled cylindrical hole array fabricated within the p-GaN contact layer of the PhC LED, as shown in Fig. 7a. In this study, the ratio of the transverse-electric and transverse-magnetic modes was approximately 7:3. This assumption was considered for an emission wavelength of 304 nm in AlGaN MQWs. Table 1 illustrates the structural parameters of nanoPSS and LED. Regarding the usual FSS-based LED (without nanoPSS and PhC; sample A), the UV light from the QWs propagates equally in all directions, as shown in Fig. 5a. Conversely, the major portion of light is absorbed by the bulk p-GaN contact layer. To avoid much light absorption by the bulk p-GaN contact layer, the emitted light from MQWs can escape vertically (guided) through the PhC in the p-GaN contact layer, as shown in Fig. 8a,b.

**Table 3:** PhC pattern size in p-GaN contact layer or p-AlGaN contact layer.

Parameter	Dimension
R/a	0.20–0.45
Order of diffraction (m)	3 and 4
D	192 nm
A	291 nm
H	140 nm
L_QP_	70 nm

Figure 8a,b shows the calculated cross-sectional electric-field (E-field) mappings in the PhC of the 304 nm UVB LEDs with nanoPSS and PhC, using the FDTD method. The cross-sectional electric-field (E-field) mappings confirm that using a PhC, high light reflectivity with regular and uniform distribution from the MQWs can be obtained, as shown in Fig. 8b. Hence, the cross-sectional E-field mappings also confirm that using the Hole-like nanoPSS and cylindrical PhC in the p-GaN contact layer, high light extraction of the UVB light can be obtained from the MQWs of UVB LEDs (Ga-LED), as shown in Fig. 8a,b.

**Fig. 9:**
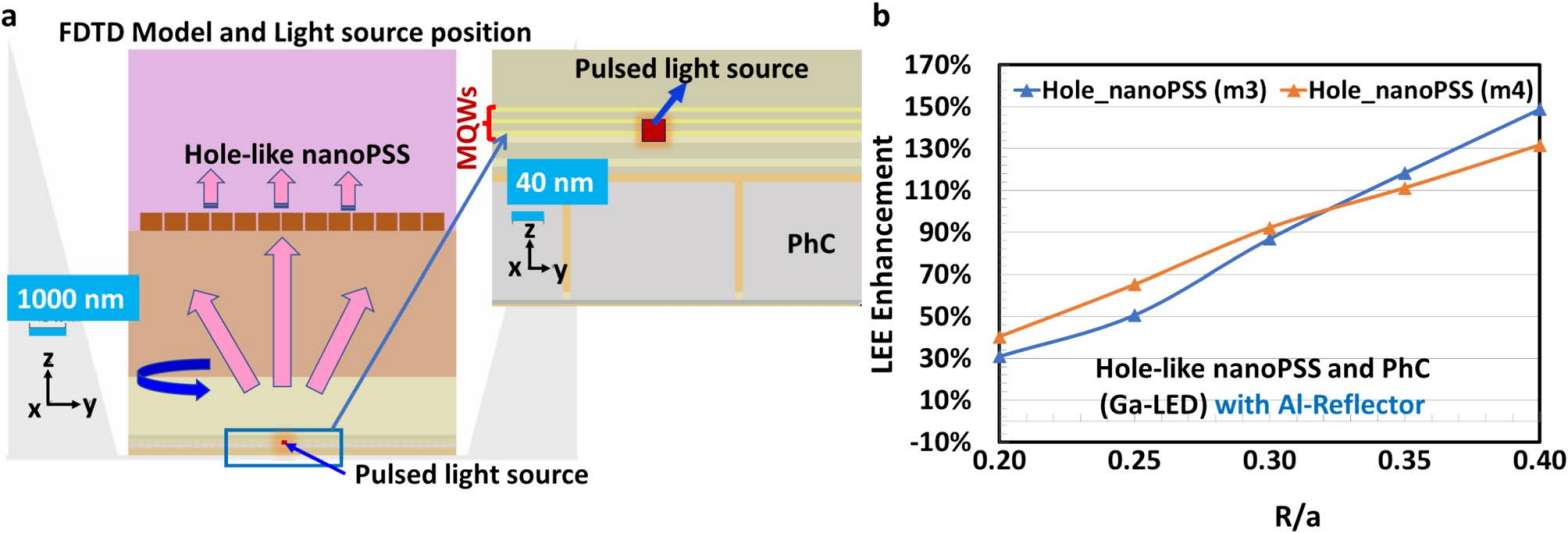
(**a**) FDTD Model and light source position along with an Al-side reflector (Ga-LED) and (**b**) LEE enhancement variation in Ga-LED as a function of R/a ratio in PhC using diffraction order (m) = 3 and m = 4 and depth (h) = 140 nm and pitch (a) = 291 nm.

The emitted light from the pulsed light source placed in the MQWs travels through the whole LED structure over the time scale of a femtosecond. The Ga-LED with pulsed light source in the MQWs is shown in Fig. 9a. Figure 9b illustrates the calculated results of light extraction enhancement as a function of R/a ratio using diffraction order (m) = 3,4, hole depth (h) = 140 nm in PhC of Ga-LED by combining the simultaneous effect of nanoPSS and PhC using an Al-side reflector. The actual value of the LEE depends on the flip-chipped UVB LED or packaging design. We assumed a Ni/Au p-electrode (having a reflectivity of approximately 34%), which can be directly evaporated on the p-GaN contact layer of Ga-LED obliquely. If we use highly reflective p-electrode like Rh, the LEE enhancement could be much higher than the case of Ni/Au. The calculated values of the LEE enhancement were approximately 149% for PhC in the p-GaN contact layer of UVB LEDs when the diffraction order (m) = 3 and R/a approximately 0.4 were chosen, as shown in Fig. 9b. However, the calculated values of the LEE enhancement were slightly lower at approximately 132% for PhC in the p-GaN contact layer of Ga-LED when the order of diffraction (m) = 4 and R/a of 0.4 was chosen, as shown in Fig. 9b. Figure 9b illustrates that the light extraction enhancement factor is the highest when the distance (L_QP_) of approximately 70 nm between MQWs and PhC is chosen. Owing to the vertical resonant Bragg’s condition for the highest possible reflection from the PhC, the maximum light extraction occurs at L_QP_ = 70 nm^24,33^. The light extraction significantly depends on the R/a values of the PhC and nanoPSS of Ga-LED, respectively. This enhanced optical performance of Ga-LED is attributable to the optimised hole-like nanoPSS (N-LED) and using highly reflecting PhC (Ga-LED).

Various reasons for the overwhelming light propagation of photons toward the p-side or escaping of photons from the active region are possible. Using optimised Hole-like nanoPSS and a new design of cylindrical-type PhC in the p-GaN contact layer is suitable for enhancing the light extraction from MQWs of the experimental UVB LEDs and LED module after evaporating highly reflective Rh or Ru p-electrode obliquely on the p-GaN contact layer of PhC. High IQE in this new design is expected after reducing the TDs in the MQWs via nanoPSS structure in the UVB LED.

Next, we examine the simultaneous influence of Hole-like nanoPSS and PhC in the highly transparent p-AlGaN contact layer of Al-LED on LEE enhancement with and without an Al-side reflector.

### Simultaneous influence of nanoPSS and photonic crystal in transparent p-AlGaN contact layer on the optical performance of UVB LED

**Fig. 10:**
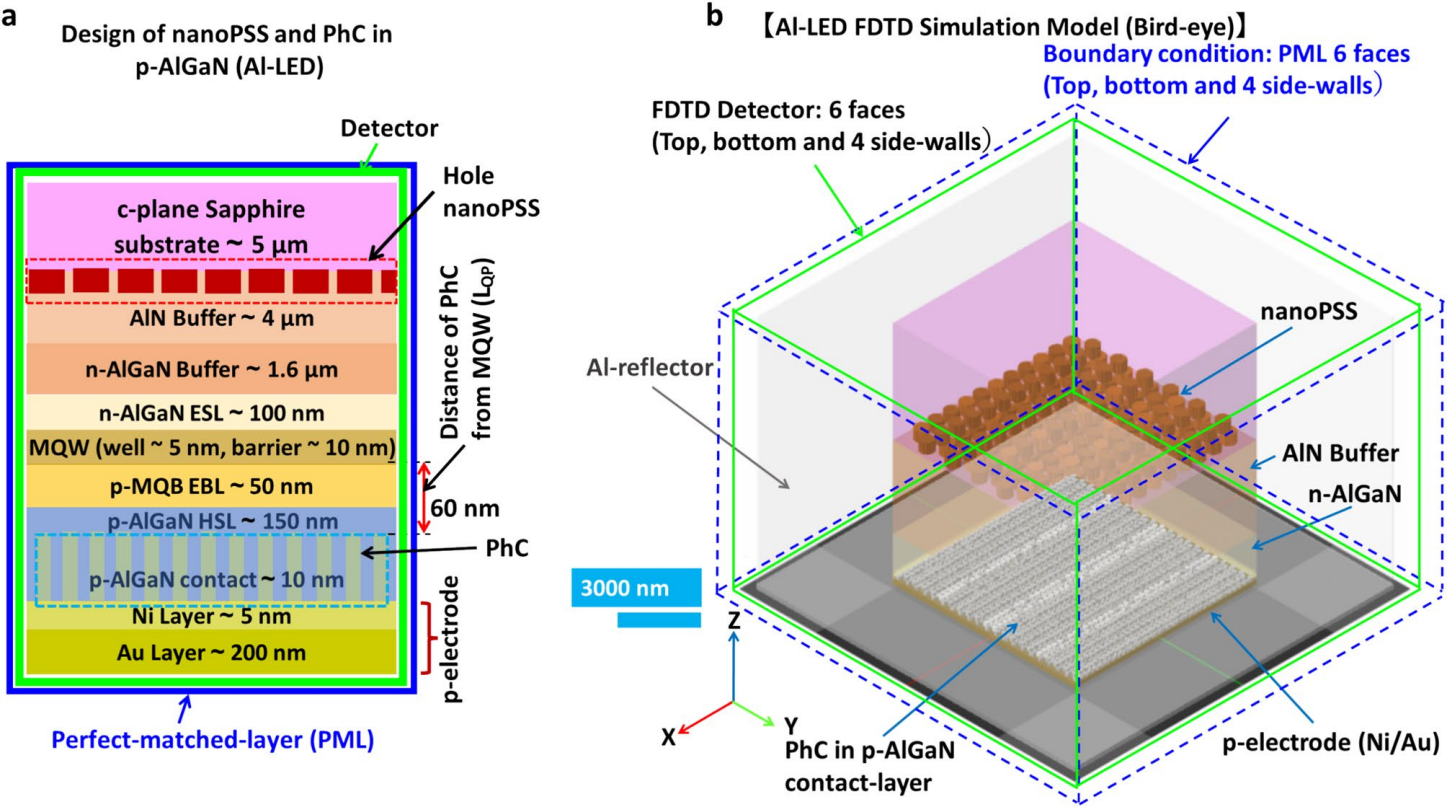
(**a**) Schematic computational models for flip-chipped Al-LEDs, where nano-patterned hole distribution in the c-plane Sapphire substrate and PhC in the highly transparent p-AlGaN contact layer are shown, and (**b**) 3D view of the Al-LED with nano-patterned hole distribution in c-plane Sapphire substrate and PhC in the p-AlGaN contact layer including the PML condition and detectors around the six faces.

**Fig. 11:**
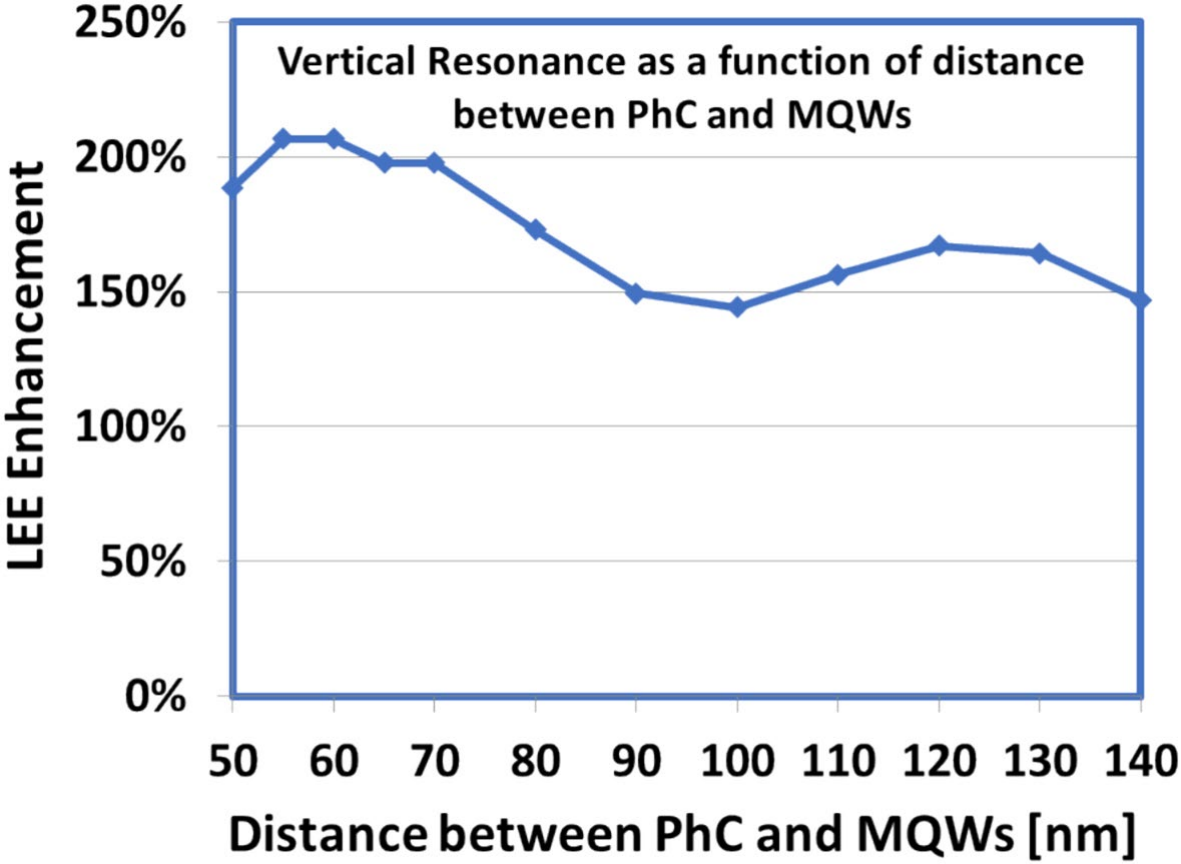
LEE Enhancement factor during vertical resonant condition as a function of distance between PhC and MQWs with standing wave stimulated by lateral Bragg’s resonance in a 2D PhC fabricated near the MQWs.

To exploit the transparent part of the p-AlGaN contact layer between the PhC structure of the p-AlGaN contact layer and PhC for light extraction enhancement, we introduced a unique design of nanoPSS and PhC in UVB LED (Al-LED), as shown in Fig. 10a,b. However, the AlN mole fraction of 70% in the p-AlGaN contact layer were the critical limit for our etching process for UVC LED^12^. However, regarding the AlGaN-based UVB emitters with AlN mole fraction of 45–50%, the etching of the p-AlGaN contact layer to fabricate a highly reflective and transparent PhC structure might be seamless and more promising compared to the UVC region. Therefore, we focused on the transparent PhC structure in UVB LED. Regarding the PhC investigation, the simulation models for the UVB LEDs are similar to Ga-LED, except using a p-AlGaN contact layer (Al-LED) instead of a p-GaN contact layer (Ga-LED). This study examined the simultaneous influence of highly reflecting PhC periodic structure in the highly transparent p-AlGaN contact layer. This includes the best optimised Hole-like nanoPSS (obtained from Section"Influence of the microPSS and nanoPSS on the LEE enhancement in UVB LED") on the optical performance of 304 nm UVB LED (Al-LED) either using a side Al-reflector or without an Al-side reflector, as shown in Fig. 10a,b. The total thickness of the p-AlGaN HSL and p-AlGaN contact layer was set to 160 nm as given in the original LED (N-LED) and shown in Fig. 10a. The PhC is set within the p-AlGaN contact layer and p-AlGaN HSL to realise a more transparent Al-LED structure. To obtain high reflectivity from PhC for UVB radiation, we tuned the periodicity, pitch (a), diameter (d), and depth (h) of the PhC, as illustrated in Table 3. However, only the distance between the PhC and the last layer of MQWs in Al-LED (L_QP_) can vary between 50 and 140 nm for examining resonant conditions, as shown in Figs. 10a, and 11. The distance between MQWs and PhC (L_QP_) changes as the PhC height changes, as shown in Figs. 10a and 11. Figure 10b shows the PML boundary condition at approximately six faces and the FDTD detector at approximately six faces, including all critical parts of the AL-LED design. We examined the LEE enhancement factor during vertical resonant conditions as a function of the distance between PhC and MQWs (distance between MQWs and PhC changes by changing the height of PhC), as shown in Fig. 11. The maximum light enhancement factor of 2.07 was obtained for L_QP_ at approximately 60 nm between PhC and MQWs in Al-LED, shown in Fig. 11, and Fig. 10a. Therefore, the position of the PhC structure is set at 60 nm from MQWs, as shown in Fig. 10a,b. The vertical resonant conditions for the highest possible reflection from the PhC may occur around this value of L_QP_ at approximately 60 nm from the MQWs, as shown in Fig. 11. The remaining structure of the Al-LED is similar to that used for the N-LED, as shown in Fig. 3. Figure 10b shows the enlarged portion of the simulated part of the transparent PhC, including the xyz-orientation. Tables 1, 2, and 3 illustrate the nanoPSS parameters, including R/a ratio, order (m), depth (h), diameter (d), and period (a), respectively, used in this part of the simulation.

**Fig. 12:**
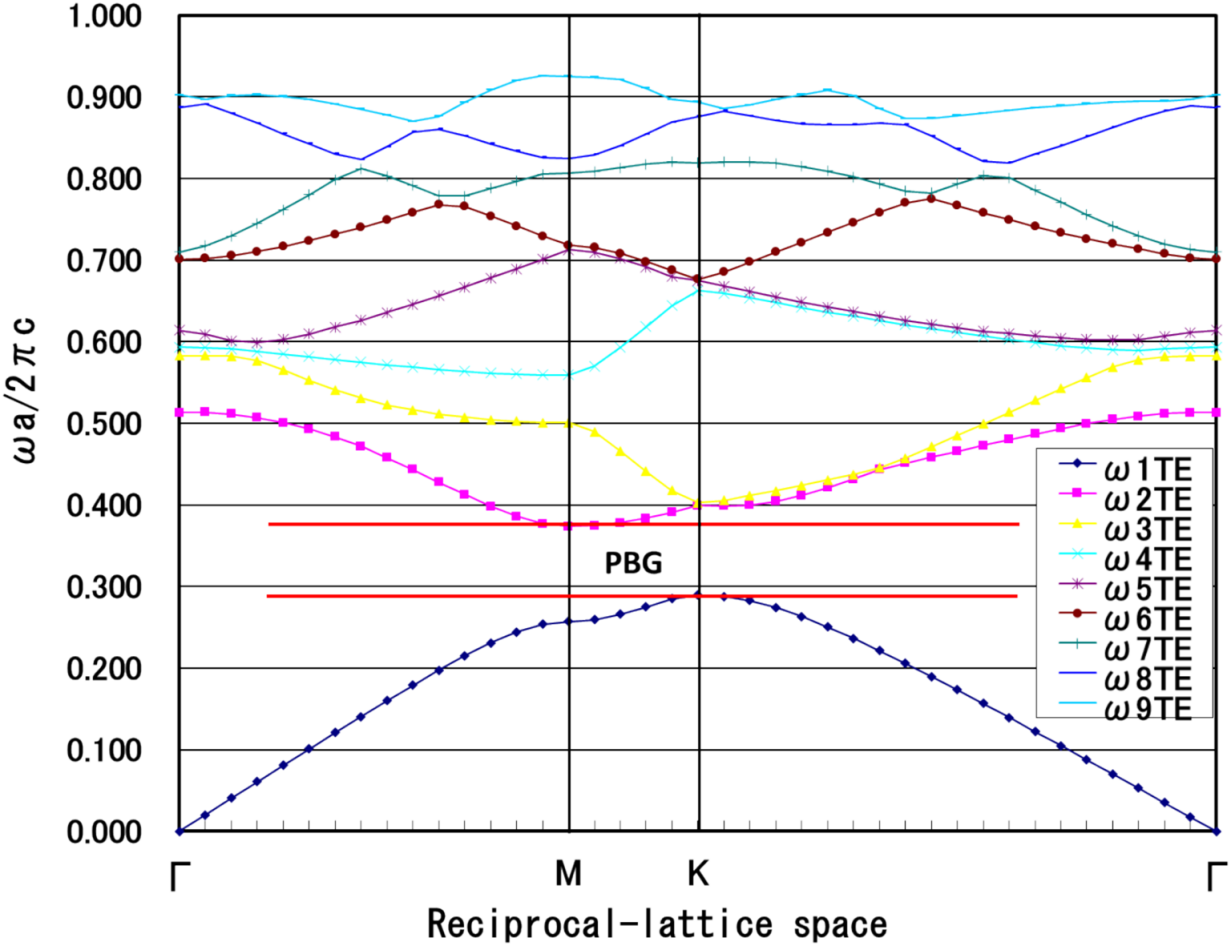
Band diagram of photonic crystal structure of circular hole on the surface of p-AlGaN PhC, including the photonic-band gap of the hole-type nanoPSS structure (Al-LED).

The PWE method^31^ was employed to confirm the photonic band gap structure and FDTD analysis by following Bragg’s condition given in Eq. (1), where *n*_*eff*_ is the effective refractive index of the PhC, and ‘a’ is the lattice period or pitch of PhC. We assumed an air Hole-type PhC with a hexagonal configuration in the p-Al_0.5_Ga_0.5_N contact layer. In this new design of PhC in the p-AlGaN contact layer, a large photonic bandgap (PBG) was observed, as shown in Fig. 12. We optimised R/a = 0.4, where R is the radius of the Holes-like structure in PhC, as illustrated in Table 3. In this study, the approximate values used for the PhC design in the p-AlGaN contact layer are λ = 304 nm, n_eff_ = 3, an diffraction order (m) = 3, 4, and pitch (a) = 291 nm. In this work, we did not use the fundamental mode of diffraction order (m) = 1 because the lattice constant ‘a’ for this mode was insignificant to fabricate. We performed more precise tuning of the air-hole period and the distance between the MQWs and PhC, as shown in Fig. 10a Vertical emission can be obtained from the standing wave simulated by lateral Bragg’s resonance condition in a 2D PhC fabricated near the last quantum well of MQWs^12,32,33^. One-directional vertical emission can be obtained by satisfying the lateral Bragg’s conditions by locating the MQWs emitter at a specific position of L_QP_ of approximately 60 nm near the PhC, as shown in Fig. 11^12,32,33^. Moreover, the optical band diagram demonstrates that good optical bandgaps for TE-polarisation modes exist in the newly designed structure of highly transparent nanoPSS and PhC in the p-AlGaN contact layer of UVB LED (Al-LED), as shown in Fig. 12.

**Fig. 13:**
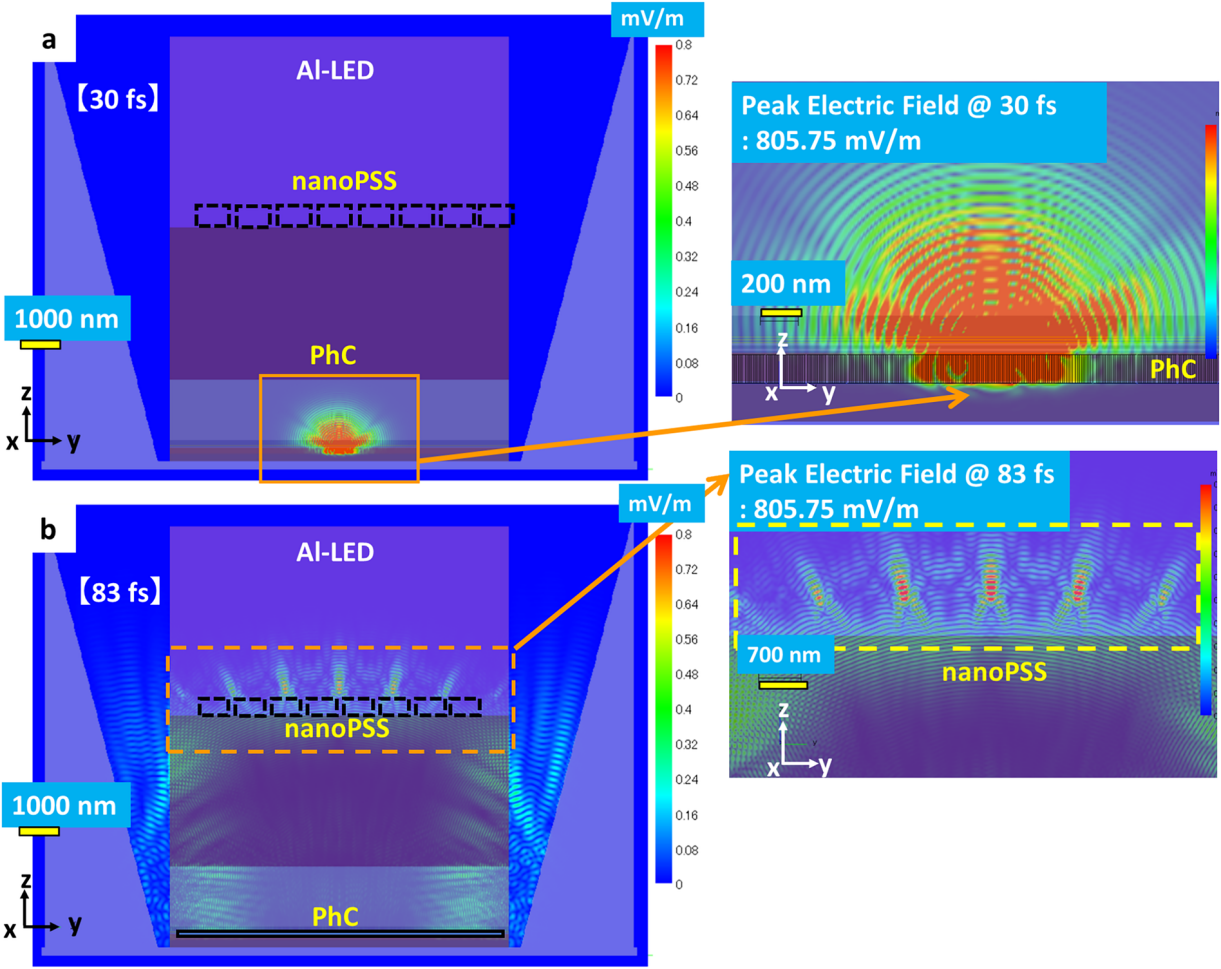
Side view of temporally simulated UVB LED (Al-LED) using FDTD model, where the magnified pulsed light source position in the MQWs and then travelling to the nanoPSS via PhC is shown in the inset. (**a**) Cross-sectional E-field mappings of 304 nm UVB LEDs (Al-LED); similar Hole-like nanoPSS and PhC and enlarged cross-sectional E-field mappings at 30.02 fs are shown in the inset, and (**b**) Cross-sectional E-field mappings of similar 304 nm UVB LEDs (Al-LED) and enlarged cross-sectional E-field mappings at 83.80 fs.

Figures 13a,b illustrate the simultaneous influence of the single pulsed source (temporal behaviour) of the simulated UVB LED (Al-LED) with Hole-like nanoPSS and PhC using the FDTD model during the short pulse operation time of the order of femtosecond (fs). The pulsed light source is placed in the MQWs region of the UVB LED, similar to Ga-LED, as shown in Fig. 9a. In our FDTS simulation model, the pulsed light source is emitted only once, as shown in Fig. 9a. The position of the pulsed light source is placed at one point close to the p-AlGaN layer of the MQW. Additionally, we examined the temporal behaviour of the emitted light via PhC and nanoPSS of Al-LED in the time range of 16 fs to 145 fs, as shown in Fig. 13a,b, which illustrates the cross-sectional E-field mappings of 304 nm UVB LEDs (Al-LED) with Hole-like nanoPSS and PhC. The temporal behaviour of the reflected light via PhC and nanoPSS in Al-LED was recorded in the Supplementary video and the same behaviour has been shown in Supplementary Figure S5.

The emitted light from this pulsed light source travels through the whole LED structure over the time scale of a femtosecond. For example, at 30 fs, the light reaching the PhC is reflected by the PhC structure, as shown in Fig. 13a. When more time has passed to 83 fs, the propagated light is transmitted through the nanoPSS, as shown in Fig. 13b. PhC and nanoPSS cannot emit light at the same fs time scale during the pulsed operation because the observation was recorded under only a single pulse emission, not under continuous operation. However, during continuous operation, high LEE in the experimental UVB LED is expected using such novel nanoPSS and PhC structures in Al-LED. We set the spatial resolution for the x–y direction at 10 nm in these simulation models, and more information about such choice is illustrated in Supplementary Figure S6a-d.

**Fig. 14:**
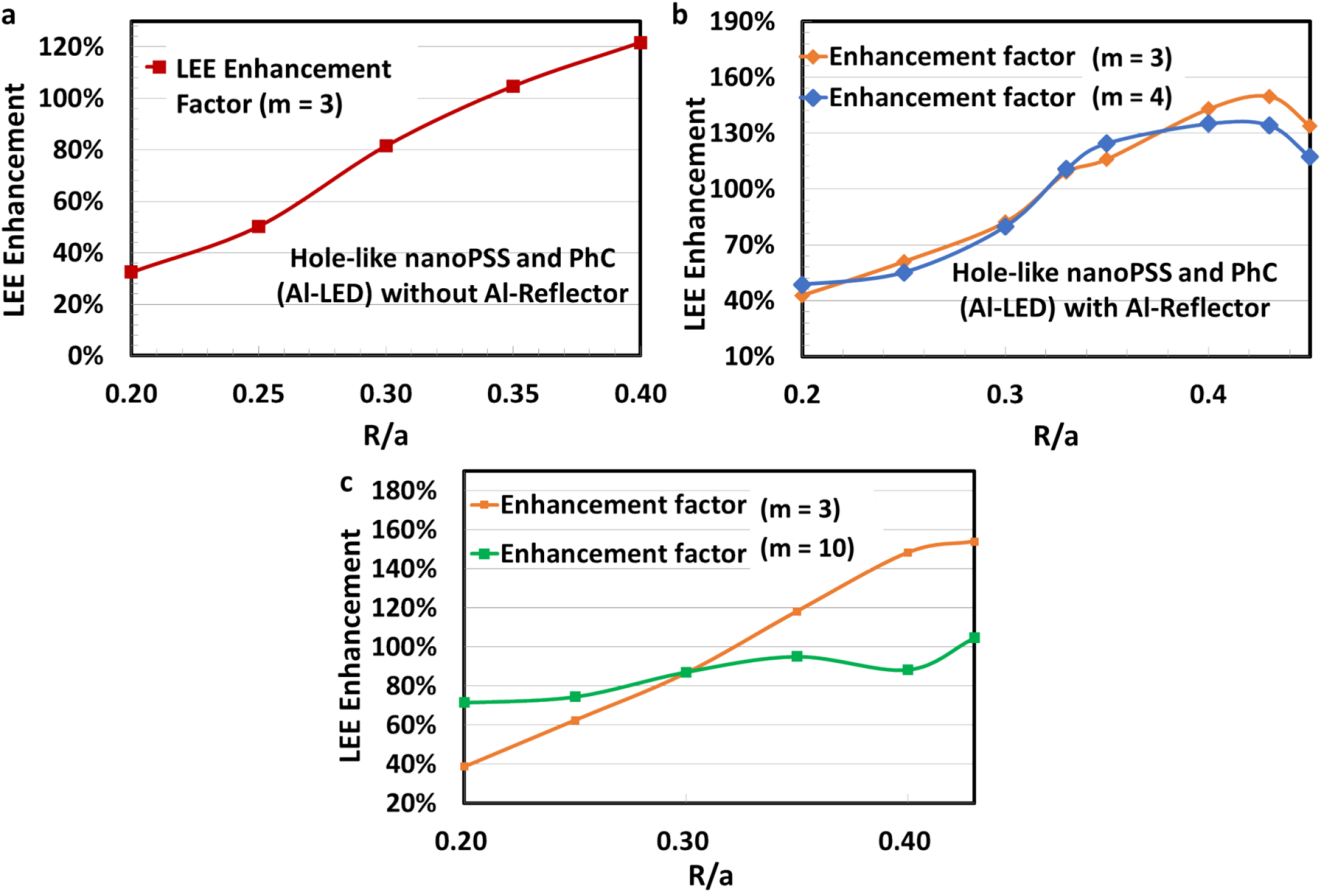
LEE enhancement variation as a function of R/a ratio using diffraction order (m) = 3, 4 and depth (h) = 150 nm and of pitch (**a**) = 291 nm in (a) Al-LED (without Al-side reflector), (**b**) Al-LED (with Al-side reflector), and (**c**) LEE enhancement variation in Al-LED as a function of R/a ratio using order of diffraction (m) = 3,10 with an Al-side reflector.

This study also examined the simultaneous influence of nanoPSS and PhC in a transparent p-AlGaN contact layer without using an Al-side reflector. Figure 14a shows the calculation results of light extraction variation as a function R/a ratio using diffraction order (m) = 3, depth (h) = 140 nm, and pitch (a) = 291 nm in the transparent Al-LED (without an Al-side reflector), demonstrating the simultaneous effect of nanoPSS and PhC on the optical performance. We assumed a Ni/Au p-electrode (reflectivity of approximately 34%), which can be directly evaporated obliquely on the PhC in the p-AlGaN contact layer of UVB LED. The calculated values of LEE enhancement of approximately ∼ 120% (without an Al-side reflector) were achieved for PhC in the p-AlGaN contact layer of Al-LEDs using diffraction order (m) = 3 and R/a of 0.4, as shown in Fig. 14a. Figure 14b illustrates the calculation results of light extraction variation as a function of the R/a ratio using the diffraction order (m) = 3 and 4, depth (h) = 140 nm, and pitch (a) = 291 nm in the transparent Al-LED (with an Al-side reflector), examining the simultaneous effect of nanoPSS and PhC on the optical performance. The calculated value of the LEE enhancement of approximately ∼ 145% was achieved for PhC in the p-AlGaN contact layer of Al-LEDs using diffraction order (m) = 3 and R/a approximately ~ 0.4, as shown in Fig. 14b. The Al-side reflector yields LEE superior enhancement of 25% than that of using no Al-reflector in Al-LED, as shown in Fig. 14b. Similarly, the calculated values of the LEE enhancement for PhC in the p-AlGaN contact layer of Al-LEDs are higher at approximately ∼ 150% compared to the Ga-LED (with an Al-side reflector) when the diffraction order (m) = 4 and R/a = 0.4 is used. However, a decreasing trend in the light extraction is observed beyond the R/a = 0.43 value, as shown in Fig. 14b. By increasing the R/a ratio up to 0.45 in the Al-LED, the LEE enhancement of approximately ∼154% at R/a = 0.43 value was observed (between R/a = 0.40 and 0.45) despite the fabrication of such R/a = 0.43 being challenging exceeding R/a = 0.40 cannot be fabricated in the practical devices owing to our limitation of available technology nodes. For example, for the scenario of PhC with the size of R/a = 0.45, for the diffraction order (m) = 3 at an emission wavelength of 304 nm, a pitch (a) = 293 nm and diameter (d) = 264 nm are crucial, indicating that the distance between any two adjacent hole patterns of PhC is restricted to only 29 nm. However, this narrow PhC is impossible by using our current technology node. Therefore, passivation remedies for the damaged surfaces caused by etching during the fabrication of an insignificant size of PhC are required. In these findings, the PhC in the p-AlGaN contact layer with R/a = 0.40 was assumed to be etched off for experimental purposes with moderate Al-contents of 45–50% for UVB emitters. This design, including nanoPSS (N-LED), will help to practically enhance the LEE and suppress the TDs in the transparent AlGaN UVB LEDs (Al-LED) to obtain high IQE.

This study also examined the influence of high diffraction order (m) = 10 in Al-LED and compared it with lower diffraction order (m) = 3, using an Al-side reflector. In our simulation strategies, we set the spatial resolution for the x–y direction at 10 nm, examining the influence of a higher diffraction order (m) on the light extraction efficiency. By discretisation, the roughness of the circular shape of PhC was different for m = 10 and m = 3, respectively, as shown in Supplementary Figure S6a-d. Table 4 and Table 5 show the simulation condition for diffraction order (m) = 10 and PhC sizes. Consequently, the LEE enhancement factor for a higher diffraction order (m) = 10 was less significant than the diffraction order (m) = 3 using R/a = 0.40, as shown in Fig. 14c. Therefore, m = 3 or 4 is suitable for the PhCs pattern in the transparent UVB LED devices to get the maximum LEE.

**Table 4:** Simulation condition for higher diffraction order (m) = 10.

Condition	
LED structure	p-AlGaN contact (150 nm + 10 nm)
nanoPSS	Hole, R/a = 0.40, m = 10, h = 500 nm
PhC	Hole, R/a = 0.20 ~ 0.43, m = 10, h = 150 nm

**Table 5:** Different PhC sizes.

R/a	d (nm)	Pitch (nm)
0.20	514	1284
0.25	669	1338
0.30	848	1414
0.35	1066	1522
0.40	1348	1685
0.43	1571	1826

This study demonstrates that several good reports exist where the advantages of either nanoPSS or PhC were reported and recommended. However, the simultaneous influence of the nanoPSS and PhC on the light extraction efficiency of UVC and UVB LEDs has not been studied before. The simultaneous influence can potentially increase the IQE, LEE, and EQE of the UVB emitters. Therefore, using reasonably optimised Hole-like nanoPSS with a new design of PhC either in the p-GaN contact layer or in the p-AlGaN contact layer of approximately 60–70 nm from MQWs is suitable for LEE enhancing of UVB LED to approximately 154% (Al-LED). Two choices are available: Either Ga-LED or transparent Al-LED in the perspective of their electrical or optical advantages. A trade-off exists between picking either Al-LED with and without an Al-side reflector in transparent Al-LED, considering device sizes or optical advantages. Significantly high LEE is expected in the Al-LED if the electrical contact on the top of PhC is properly engineered in the flip-chipped Al-LED.

Therefore, a new design of optimised Hole-like nanoPSS and PhC in the p-AlGaN contact layer of UVB LED may also be advantageous for the flip-chipped researchers interested in high light power devices. This includes WPE in the UVB module technology of watt-class, where EQE of 10% and light power of 42 mW on the wafer in a single 304 nm UVB LED was already reported^34^. We need the optimization of Mg-dopants in UVB LEDs to enhance the LEE^35^. The resulting periodic structure has a sufficiently large photonic-crystal band-gap effect for the possibility of high efficiency in transparent UVB-LED application. This is crucial in the development of high-power UVB-LED modules with high WPE suitable for immunotherapy (psoriasis, vitiligo), vulgaris treatment (310 nm), plant growth under UVB lightning (310 nm), the production of vitamin D_3_ in the human body (293–304 nm), the production of phytochemicals in green leaves of vegetables (310 nm) and inactivation of SARS-CoV-2^9,10,16^.

## Summary

This study employed the FDTD and the PWE methods to examine the simultaneous influences of nanopatterned sapphire substrate (nanoPSS) and photonic crystal (PhC) either in the p-GaN contact layer or in the transparent p-AlGaN contact layer on the optical power emission and light extraction in flip-chipped AlGaN-based UVB LEDs at 304 nm emission wavelength. The Hole-like nanoPSS was found to be suitable after optimising the pitch (a), diameter (d), depth (h), and R/a ratio of nanoPSS in UVB LEDs, compared to the microPSS or Pillar-like nanoPSS at the emission wavelength of 304 nm. Introducing cylindrical Hole-like nanoPSS with a diameter (d) = 596 nm, pitch (a) = 746 nm, height (h) = 500 nm, and R/a = 0.38, along with an order of diffraction (m) = 10 and a side Al-reflector, significantly enhanced the light extraction by approximately 18% compared to the microPSS or Pillar-like nanoPSS in the UVB LED without using PhC. Finally, two cases for a simultaneous influence of an optimised nanoPSS and PhC on theoretical light extraction in flip-chipped AlGaN-based UVB LED using PhC in p-GaN contact layer PhC in p-AlGaN contact layer were examined. First, we examined the influence of not using a side Al-reflector on light extraction, which resulted in a lower enhancement of approximately 120% for PhC in the p-AlGaN contact layer of Al-LEDs. Subsequently, we examined the effect of cylindrical Hole-shaped nanoPSS with the order of diffraction (m) = 3 and R/a ratio of approximately 0.4. Finally, by simultaneously introducing the new hole-like nanoPSS and PhC (Hole-like) in the p-GaN contact layer or the p-AlGaN contact layers, using R/a = 0.40 and height = 150 nm, with a side Al-reflector in the UVB LED, the light extraction was enhanced to 132% (p-GaN contact layer) and 145% (p-AlGaN contact layer) using an order of diffraction (m) of approximately 4. Increasing the R/a ratio to 0.45 in the Al-LED yielded one additional high peak of light extraction of 154% at R/a = 0.43 (between R/a: 0.40 and 0.45). The study results demonstrated that practical devices are unsuitable for fabricating PhC size using R/a = 0.40 owing to the limited availability of technology nodes at Riken. This is the highest value of light extraction enhancement reported in the optically simulated UVB-LED and for the experimental UVB LED. UVB LED modules technologies of Watt-class and sub-Watt-class have potential applications, such as those in immunotherapy (psoriasis, vitiligo), vulgaris treatment (310 nm), plant growth under UVB lightning (310 nm), the production of vitamin D_3_ in the human body (293–304 nm) and phytochemicals in green leaves of vegetables (310 nm), and inactivation of SARS-CoV-2.

## Supplementary Information

Supplementary Information 1.

Supplementary Video 1.

## Data Availability

The data supporting this study’s findings are available from the corresponding author upon reasonable request.
