# Supplementary material for: Simultaneous influence of nanoPSS and photonic crystal on light extraction in AlGaN 304nm UVB LEDs
Source: Sci Rep. 2025 Jun 6;15:19972. doi: 10.1038/s41598-025-03746-6 (PMC12144251; doi:10.1038/s41598-025-03746-6)
Supplement: Supplementary file 1 — Supplementary Information 1. [file 41598_2025_3746_MOESM1_ESM.docx]

# **Optimisation of microPSS and nanoPSS for Efficient Light Extraction and** **Temporal Behaviour of the Pulsed AlGaN UVB LED with p-AlGaN Contact-Layer**

M. Ajmal Khan^1, *^, E. Matsuura^1^, Y. Kashima^1^, H. Hirayama^1^

*^1^RIKEN Cluster for Pioneering Research (CPR), 2-1 Hirosawa, Wako, Saitama 351-0198,*

*Japan*

Herein, we aim to explore the best microPSS or nanoPSS to be coupled with highly reflective photonic-crystal (HR-PhC) in UVB LED for maximum possible light extraction. This supplementary note discusses and compares the FDTD simulation results of microPSS and nanoPSS-based UVB LEDs, especially the emitted power, LEE enhancement, and cross-sectional electric-field (E-field) mappings. Finally, we simulated and examined the most refined simulation results of nanoPSS and HR-PhC on the UVB-LED performances of this study. Moreover, it examines the detailed temporal behaviors of the optical light propagation in pulsed AlGaN UVB LED with p-AlGaN contact layer (Al-LED). The first part outlines the optical modeling design structure, parameters, and results of microPSS.

# **I). Comparative Study of microPSS FDTD Simulation Model in UVB LED**

Herein we simulated two different types of microPSS structures, either having a (Cone+Dome)-like shape (sample B) or a Pyramid-like shape (sample C) to see the influence on power and LEE enhancement in UVB LED, and all parameters and results are illustrated in Table S1. Table S1 illustrates

Table S1: Results of FSS-based LED (sample A), (Cone+Dome)-like shape (sample B), and Pyramid-like shape (sample C) microPSS on the power and LEE enhancement of UVB LED.

| **sample /(R/a)** | **Pattern size** | **Power [arb. u]** | **LEE Enhancement** |
| --- | --- | --- | --- |
| sample A /N.A | Flat c-plane sapphire substrate (FSS) | 6.13×10^-17^ | - |
| sample B/0.33 | diameter (d ) = 2.0 µm, height (h) = 1.5 µm, and pitch (a) = 3 µm | 6.59×10^-17^ | 7 % |
| sample C/0.42 | d = 2.5 µm, h =1.5 µm, and a = 3 µm | 6.45×10^-17^ | 5 % |

the results of sample B and sample C, where approximately ≈ 5-7 % of LEE enhancements are observed. Consequently, (Cone+Dome)-like (sample B) yielded superior LEE enhancement of approximately 7 % than the flat-surface sapphire substrate (FSS)-based LED (sample A) and sample C too.

Figure S1 shows the cross-sectional electric-field (E-field) mappings of 304 nm UVB LEDs without microPSS (sample A), with (Cone+Dome)-like microPSS structure (sample B), and with Pyramid-like microPSS (sample C), respectively. The in-plane E-field mappings are calculated at the interface between the AlN and c-plane Sapphire. Significantly high light extraction in (Cone+Dome)-like microPSS (sample B) was observed, as shown in Figure S1b. The (Cone+Dome)-like structure in UVB LED was superior to the Pyramid-like structure (sample C), as shown in Figure S1b,c.


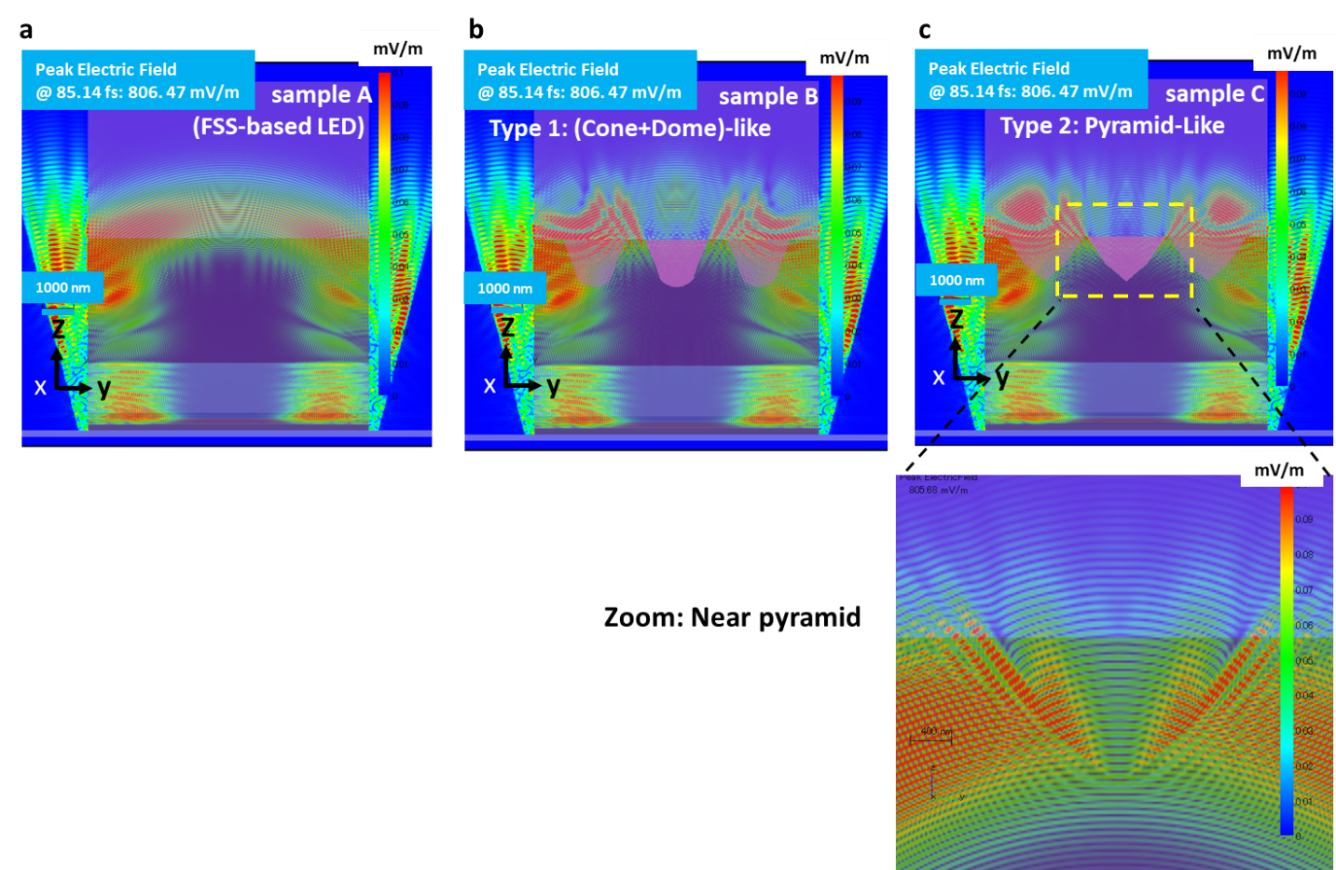


Figure S1. (Colour online) The cross-sectional electric-field (E-field) mappings of the AlGaN-based UVB LEDs (a) flat-surface sapphire substrate (FSS)-based LED (sample A), (b) with (Cone+Dome)-like microPSS structure (sample B), and (c) with Pyramid-Like microPSS structure (sample C).

In the next Part II the LEE enhancements in two types of nanoPSS in UVB LED were investigated.

**II). Investigation of** **Two Types of nanoPSS in UVB LED**

In this part, first the simulation results of FDTD model of (Cone +Dome)-like nanoPSS (sample Da) with h = 500 nm and d = 500 nm were analysed, where pitch (a) was allowed to vary between 700 nm – 1500 nm (varying R/a= 0.17- 0.33), as illustrated in Table S2. Table S3 illustrates structural information,

Table S2: Varying of pitch (a) in sample Da to get different values of R/a ratio.

| **Pitch (a)** | **R/a** |
| --- | --- |
| 750 nm | 0.33 |
| 1,000 nm | 0.25 |
| 1,500 nm | 0.17 |

emitted power, and LEE enhancement of sample Da. R/a = 0.33, h = 500 nm, a = 750 nm, and d = 500 nm exhibit superior LEE enhancements of 6 % in sample-Da, compared to the smaller R/a values of 0.17 and 0.25. These results suggest that a ~ 750 nm and R/a ~ 0.33 value dependence of nanoPSS are suitable for UVB emission, and the same is supported by cross-sectional in-plane E-field mappings, as shown in Figure S2. Subsequently, we compared these results with the microPSS given in Part I and found that the nanoPSS with given design parameters as illustrated in Table S3 is still lower than microPSS.


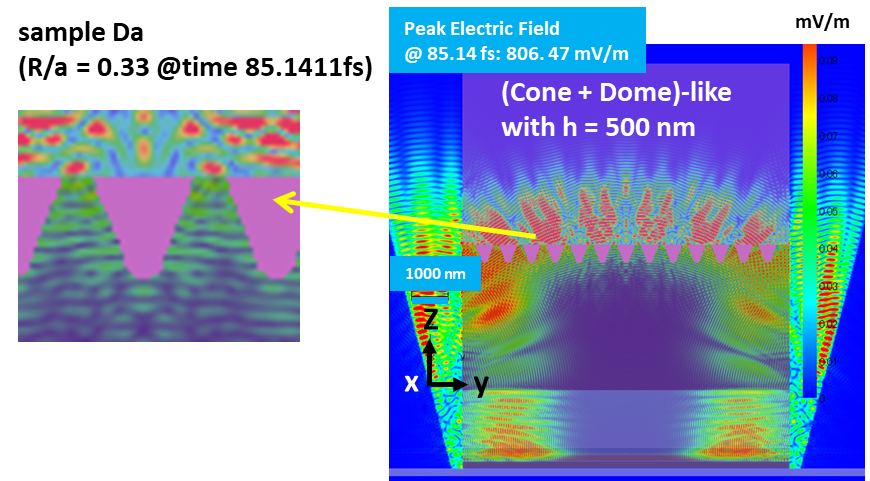


Figure S2. (Colour online) Cross-sectional in-plane E-field mappings of 304 nm UVB LEDs with (Cone+Dome)-like nanoPSS (sample Da).

Table S3: FDTD simulation results and comparison of reference FSS-based LED (sample A) with (Cone+Dome)-like nanoPSS (sample Da), where h = 500 nm and d = 500 nm, and R/a are varied.

| **sample / (R/a)** | **Pattern size** | **Power [arb. u]** | **LEE enhancement** |
| --- | --- | --- | --- |
| sample A | - | 6.13×10^-17^ | - |
| sample Da/0.33 | d = 500 nm, h = 500 nm, and a = 750 nm | 6.52×10^-17^ | 6 % |
| sample Da/0.25 | d = 500 nm, h = 500 nm, and a = 1,000 nm | 6.39×10^-17^ | 4 % |
| sample Da/0.17 | d = 500 nm, h= 500 nm, and a = 1,500 nm | 6.25×10^-17^ | 2 % |

Therefore, based on sample Da results, we further investigated the impact of much smaller and more practical (Cone+Dome)-like nanoPSS (sample Dd) with d = 300 nm and h = 300 nm on LEE enhancement, as shown in Figure S3. However, only pitch (a) was allowed to vary between 375 – 1000 nm, as illustrated in Table S4. Table S5 illustrates the simulation results of using nanoPSS with an insignificant diameter in UVB LED as a function of R/a in the range of 0.14-0.4. Finally, R/a = 0.33 with h = 300 nm, and d = 300 nm yields similar power and light extraction up to 6 % as achieved for the best sample Da, as illustrated in Table S5.

Figure S3 demonstrates the E-Field mapping for sample Dd with R/a = 0.33, d = 300 nm, a = 450 nm, and h = 300 nm @ time 85.1411 fs. The LEE enhancement of the UVB light can be high compared to the other R/a values, as illustrated in Table S5. However, the LEE enhancement is still low (6 %), when compared to microPSS (7 %) given in Table S1. However, the effect of Hole-like or cylindrical-like nanoPSS structures on the light extractions in UVB LED has not been investigated yet.

## Table S4: The condition of pitch (a) in sample Dd.

| **Pitch (a)** | **R/a** |
| --- | --- |
| 375 nm | 0.40 |
| 450 nm | 0.33 |
| 600 nm | 0.25 |
| 1,000 nm | 0.15 |


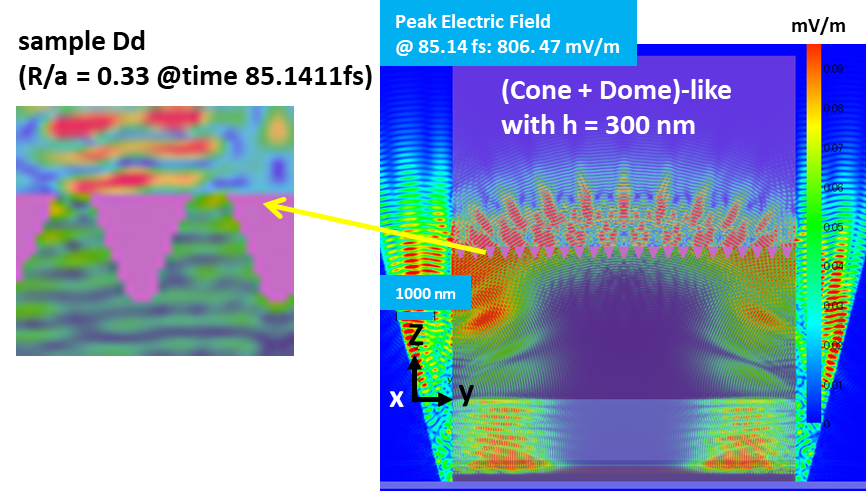


Figure S3 (Colour online) Cross-sectional in-plane E-field mappings of 304 nm UVB LEDs with (Cone+Dome)-like nanoPSS structure (sample Dd).

Table S5: FDTD parameters and results of (Cone+Dome)-like nanoPSS (sample Dd) with height = 300 nm and d = 300 nm, varying the R/a values (Pitch(a)).

| **sample / (R/a)** | **Pattern size** | **Power [arb. u]** | **LEE Enhancement** |
| --- | --- | --- | --- |
| sample A | - | 6.13×10^-17^ | - |
| sample Dd /0.40 | d = 300 nm, h = 300 nm, a = 375 nm | 6.35×10^-17^ | 3 % |
| sample Dd /0.33 | d = 300 nm, h = 300 nm, a = 450 nm | 6.49×10^-17^ | 6 % |
| sample Dd /0.25 | d = 300 nm, h = 300 nm, a = 600 nm | 6.46×10^-17^ | 5 % |
| sample Dd /0.15 | d = 300 nm, h = 300 nm, a = 1,000 nm | 6.28×10^-17^ | 2 % |

Therefore, in Part III FDTD simulation model and results of Hole-like and Pillar-like nanoPSS in UVB LED are briefly discussed and analysed.

**III). Analysis of Hole-like and Pillar-like nanoPSS in UVB LED**

Herein, we simulated and analysed the comparative performances of Hole-like (sample-E) and Pillar-like (sample F) nanoPSS structures in UVB LED to determine whether such a new optical design could improve LEE enhancement beyond 6-7 %. The height (h) = (2/3)×a was fixed, as illustrated in Table S6. However, we only varied the order of diffraction (m) between 1 and 13, as given in Table S6. The power and LEE enhancement of Hole-like nanoPSS (sample E) and Pillar-like nanoPSS (sample F) UVB LEDs as a function of diffraction order (m) for fixed R/a values are investigated. Consequently, significant

Table S6: nano-pattern sizes R/a = 0.35 and R/a = 0.38, for Hole-like nanoPSS (sample E) and nano-pattern sizes R/a = 0.38 for Pillar-like nanoPSS (sample F).


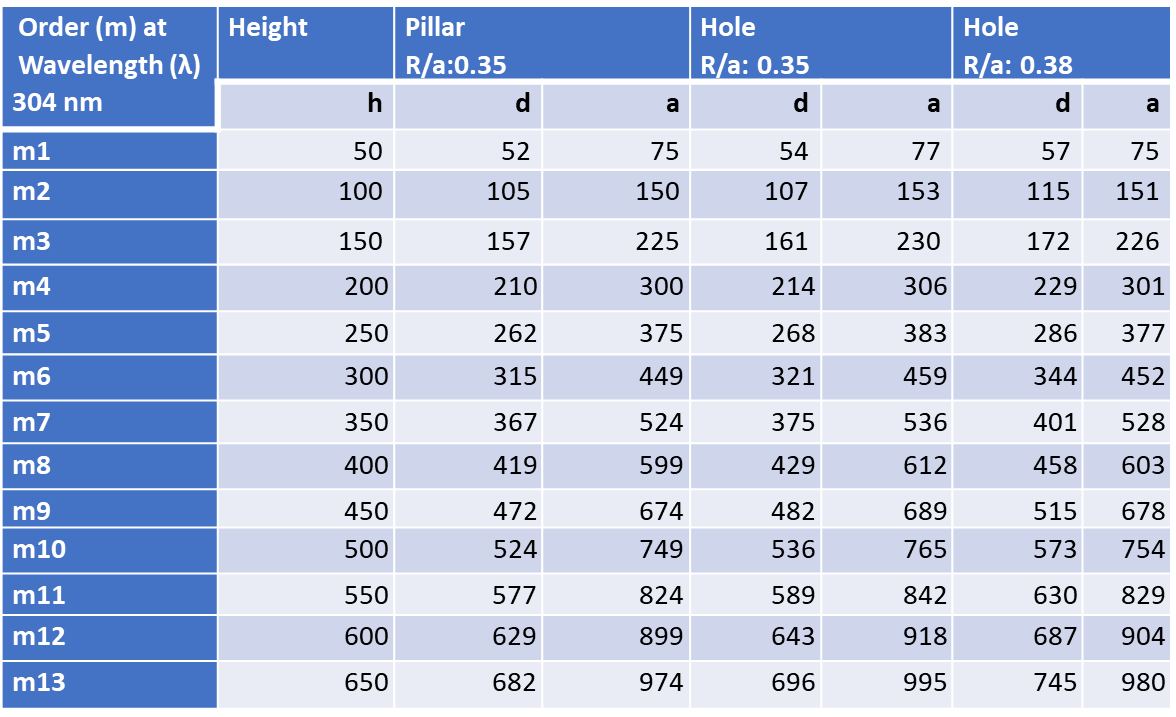


improvement in the light power of approximately ≈ 7.16 ×10^-17^ [arb. u] and LEE enhancement of ≈ 15 % in sample E using order of diffraction (m) =10 and R/a = 0.38 were observed, as shown in Figure 2g of this study. The light power and LEE enhancement in Hole-like nanoPSS (sample E) in LED with R/a = 0.35 exceeded that of Pillar-like nanoPSS (sample F), as shown in Figure 2g of the main body of the paper. This result is seemingly winning one and therefore, we further refined it in the main body of the paper.

Figure S4a-c shows the E-Field mapping of FSS-based LED (sample A) and the special case of nanoPSS-based LED with R/a = 0.35, order of diffraction (m) = 10 and h = 500 nm @ time scale of 85.1411fs in the Hole-like nanoPSS (sample E) and Pillar-like nanoPSS (sample F). The cross-sectional E-field mappings show that using Hole-like nanoPSS (sample E) is promising, where the light extraction of the UVB LED was remarkably enhanced compared to the Pillar-like nanoPSS (sample F) and sample A.

Additionally, the temporal behaviour of enhanced light propagation in the transparent Al-LEDs was investigated under pulsed conditions. Figure S5 shows the cross-sectional E-field mappings of temporally pulsed 304 nm UVB LEDs (Al-LED) with Hole-like nanoPSS and PhC under the single pulsed source


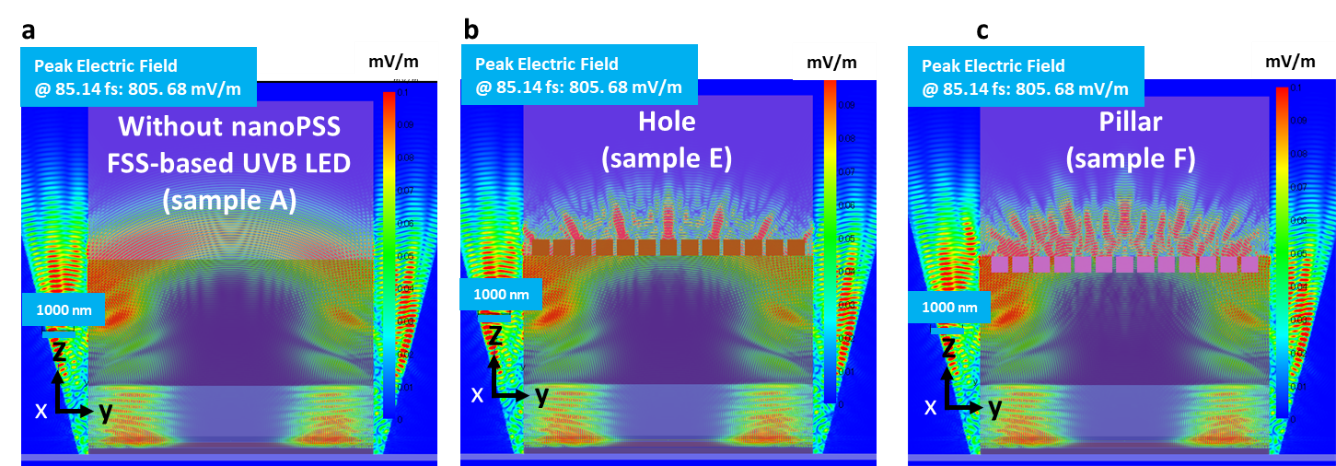


Figure S4 (Colour online) Cross-sectional E-field mappings of UVB LEDs (a). without nanoPSS (sample A), (b). with Hole-like nanoPSS (sample E), and (c). with Pillar-like nanoPSS (sample F).


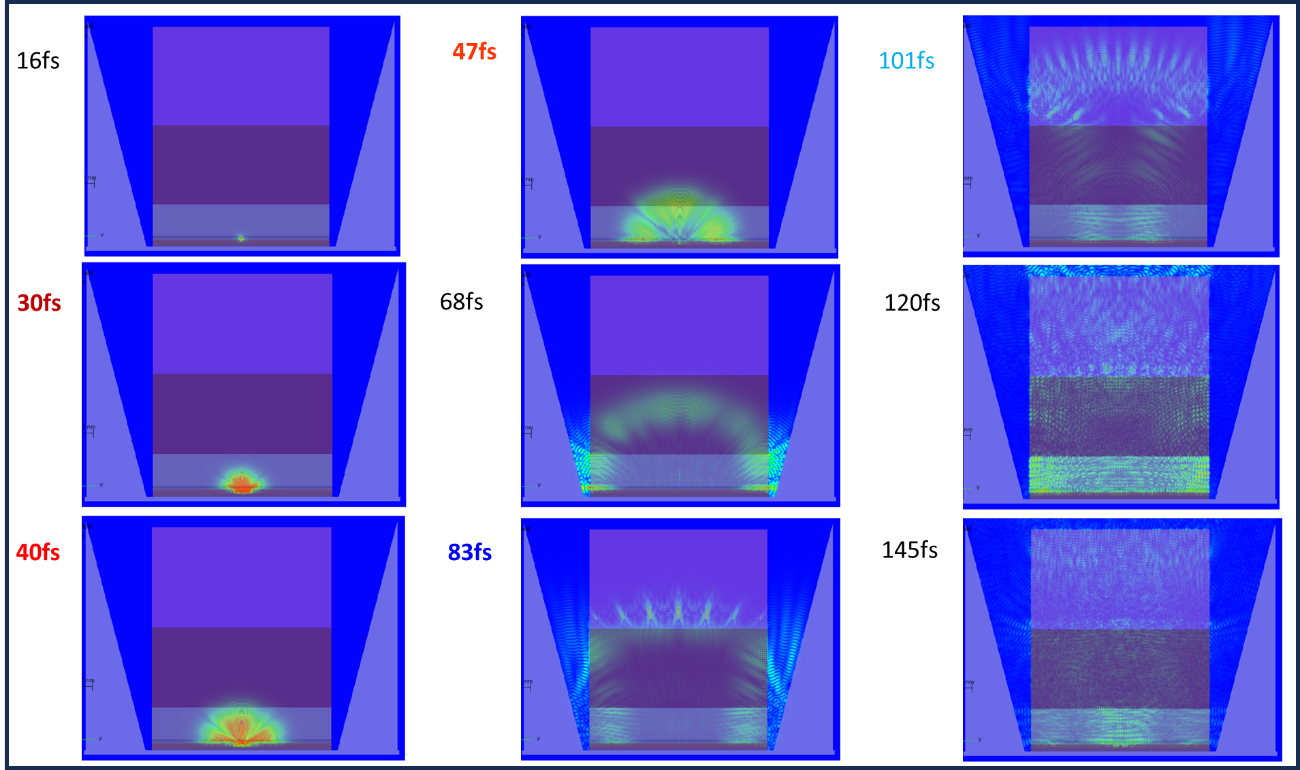


Figure S5. (Colour online) Side view of the Temporally simulated UVB LED (Al-LED) using FDTD model, where the pulsed light source is placed in the MQWs region and cross-sectional E-field mappings of 304 nm UVB LEDs (Al-LED) with Hole-like nanoPSS and PhC under the single pulsed source operation at 16 fs to 145 fs.

operation, using the FDTD model. The light extraction behaviour was monitored over the time scale from 16 to 145 fs, as shown in Figure S5. For example, at 30 fs, the light that reaches the reflective-PhC (R-PhC) is reflected only by R-PhC. In addition, at 83 fs, when further time has passed, it shows that the propagated light is transmitted through the nanoPSS only. However, R-PhC and nanoPSS are inherently incapable of emitting light instantaneously on a femtosecond time scale. This is attributable to their emission being displayed over a longer time scale under a single pulsed operation. However, experimental devices can emit light instantaneously in nanoPSS and PhC during continuous operation. The same optical light propagation dynamics have been observed in the Supplementary video.

In Part V the influence of high order of diffraction (m) for Photonic Crystals (PhC) in UVB LED (FDTD Simulation Model) on LEE enhancement is discussed.

**V). Choice of the** **High** **Order of Diffraction (m) for PhC in FDTD Simulation Model**


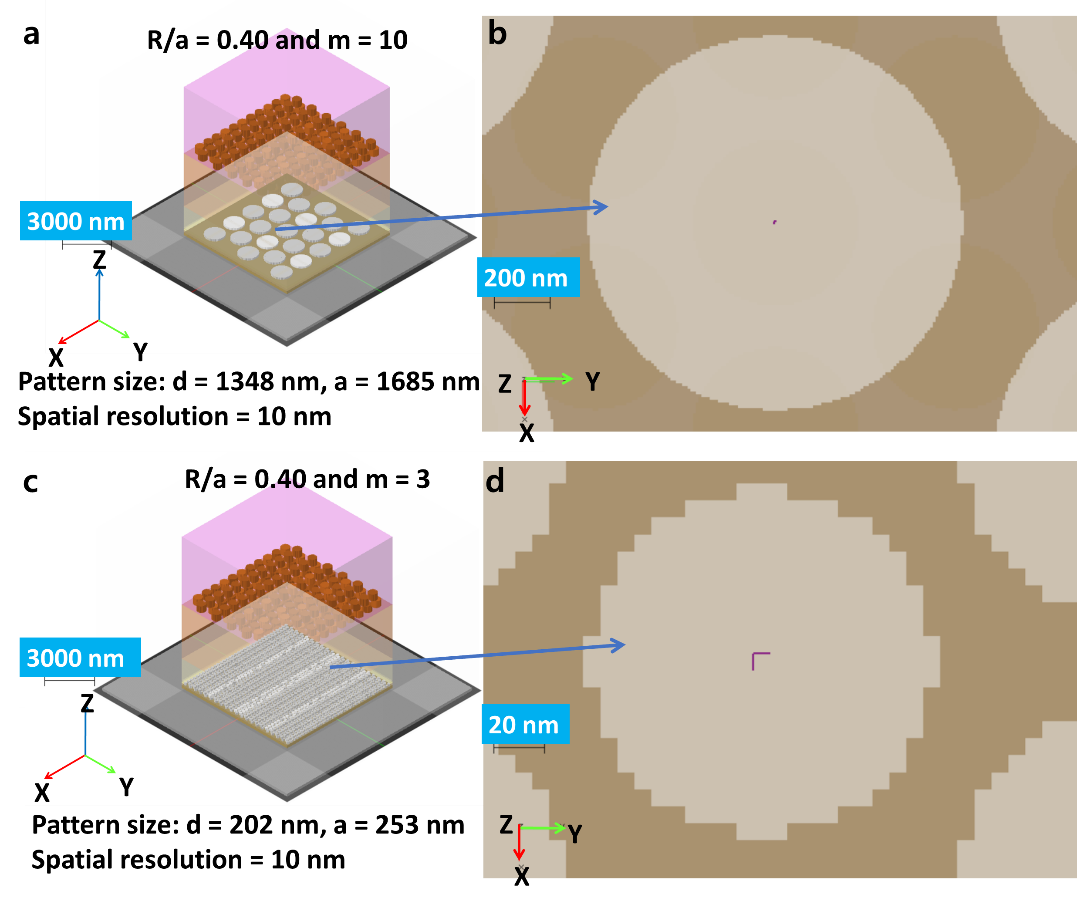
To simulate PhCs by spatially resolved approaches, such as FDTD, considering a sufficient number of

Figure S6a-b. (Colour online) Spatial resolution for x-y direction at 10 nm for pattern size (d = 1348 nm and a= 1685 nm, R/a =0.40), and (c-d) Spatial resolution for x-y direction at 10 nm for pattern size (d = 202 nm and a= 253 nm, R/a =0.40) in the FDTD simulation models.

orders of diffraction (m) exceeding 10, is crucial to achieving their optical response with adequate precision. Owing to the specified dimensions of the simulated stack, including radii and period of holes and cones, only a limited number of nanoPSS periods are encompassed within the simulated region. Consequently, may compromise the precise representation of the photonic properties of PhCs. Therefore, we set the spatial resolution in the x-y direction in our FDTD simulation models to approximately 10 nm to enhance understanding. Discretisation caused differences in PhC circular shape roughness for diffraction orders (m) = 10 and m = 3, respectively, as shown in Figure S6a-d. Simulated results indicated a lower LEE enhancement for the shape corresponding to a high diffraction number m = 10 than that of lower m = 3. Consequently, the simulation results revealed that the output power and the LEE enhancement factor were lower for m = 10 than for m = 3. Therefore, we selected m = 3 or 4 for PhC patterns in this study.
